# Trends in Australian Alcohol Consumption and Related Harms, 2010–2023

**DOI:** 10.1111/dar.70261

**Published:** 2026-10-19

**Authors:** Emma Vieira, Mia Miller, Amy Peacock, Nicola Man, Michael Livingston

**Affiliations:** ^1^ National Drug Research Institute Curtin University Melbourne Australia; ^2^ Burnet Institute Melbourne Australia; ^3^ National Drug and Alcohol Research Centre UNSW Sydney Sydney Australia; ^4^ Menzies School of Health Research Charles Darwin University Darwin Australia; ^5^ School of Psychology University of Tasmania Hobart Australia

**Keywords:** alcohol consumption, Australia, public health, trends

## Abstract

**Introduction:**

Alcohol consumption is a substantial contributor to Australia's total disease burden. Various studies have examined trends in specific measures of consumption or harm, but a comprehensive analysis of trends is needed to understand the changing impact of alcohol in Australia. The current study synthesised data across multiple national indicators to assess long‐term trends in alcohol consumption and related harms.

**Methods:**

Publicly accessible data were compiled from 2010 to 2023 (or until the most recent year available) and categorised into the following: non‐survey consumption measures (i.e., alcohol available for consumption and wastewater estimates); survey consumption measures (i.e., three nationally representative surveys); and alcohol‐related harm measures (i.e., deaths, hospitalisations, ambulance attendances, injuries, random breath tests and alcohol treatment episodes). Data were analysed using joinpoint regression analysis to assess trends and quantify annual percentage change.

**Results:**

Survey and wastewater measures of alcohol consumption generally declined from 2010 onwards, while per‐capita consumption as measured via tax and import data slightly increased. Rates of most alcohol‐related harms increased substantially over the same period.

**Discussion and Conclusions:**

Alcohol‐related harms in Australia have generally increased over the past 15 years. This trend, alongside declines in survey‐measures of consumption and relative stability in tax‐based estimates of per‐capita consumption raises potential concerns about the ongoing validity of population surveys as tools to measure consumption trends. Increasing harms also point to the urgent need for evidence‐based interventions in Australian alcohol policy.

## 1 Introduction

Alcohol consumption contributes substantially to the global burden of disease, resulting in both non‐communicable diseases such as cancer and liver disease, as well as shorter‐term harms such as accidents and injuries [1]. Rates of alcohol consumption in Australia are substantially higher than that of other Organisation for Economic Cooperation and Development countries, with 9.8 pure litres of alcohol available for consumption for every person aged 15 years and over in 2023–2024, compared to the 2023 Organisation for Economic Cooperation and Development average of 8.5 L [2, 3]. While the amount of alcohol available for consumption in Australia has fallen in recent years (i.e., from 10.9 L in 2020–2021 [2]), alcohol remains responsible for 4.1% of the total disease burden [4, 5] and results in annual social and economic costs of $A75 billion [6].

Studies of individual data sets in Australia suggest there have been notable changes in alcohol consumption and related harms in recent years. Survey data shows that the proportion of people drinking at risky levels has declined since 2004, alongside an increase in the proportion of Australians abstaining from alcohol [7]. However, these shifts appear to have been driven largely by steep declines in consumption among young people, with decreases among mid‐life and older adults of a smaller magnitude and predominantly evident for males when disaggregated by gender [7]. Conversely, data on alcohol‐attributable ambulance attendances and alcohol‐induced deaths suggest an upward trend in alcohol‐related harms since the mid‐2010s, with marked increases observed from 2020 onwards that are likely linked to the COVID‐19 pandemic [2, 8]. While some state and territory differences are present—such as the Northern Territory reporting the highest rate of alcohol‐attributable deaths in 2022 (33.6 per 100,000 people), while the Australian Capital Territory reported the lowest (17.34 per 100,000 people)—the overall direction of such trends has been broadly similar across the country [9]. While it is important to acknowledge potential underlying subpopulation differences in trends, whether by geographic region, age, gender or other characteristics, the aim of the current study is to focus on the overall trends in consumption and harms.

Tracking long‐term trends in alcohol consumption and harms is imperative to both inform and monitor public policy efforts. While Australia has a wealth of data sources, different measures can paint very different pictures [10] and it is necessary to synthesise a range of different data sources to provide a clearer picture of how use and harms are changing. Triangulating multiple indicators enables a more robust and comprehensive understanding of population‐level consumption and harms, while minimising the biases inherent in individual data sets [11]. For example, household surveys typically underestimate population levels of drinking by more than 50% [12], due to the exclusion of key groups from sampling (e.g., those without housing are automatically excluded, and these typically marginalised populations also tend to consume more alcohol than the general population [13]). While survey trends often broadly follow other measures of consumption [14], declining survey response rates highlight the need to reexamine these patterns. Similarly, the estimation of harm from administrative data relies on complex decisions around classification of outcomes. For example, diagnosis codes in hospitals have a range of potential biases [15, 16], and even mortality data can under‐estimate the true burden of alcohol [17]. Focussing on only a single measure can therefore be misleading.

Previous attempts at triangulating across sources on alcohol use and harms are largely limited to reports by the Australian Institute of Health and Welfare (AIHW) [2], who bring together some (but not all) data sources, with no formal analyses conducted to assess changes in trends over time. There has been some synthesis of findings by age group: older work in Australia and the United Kingdom has shown divergences between measures of consumption and harm [10, 18, 19], while more recent work has suggested that for youth populations, consumption and harms have declined in concert to varying degrees [20, 21]. Overall though, the lack of a recent comprehensive examination of population‐based trends in Australia indicates a need for updated analyses to understand how the impact of alcohol in Australia is changing. The present study thus assesses trends in alcohol consumption and harms using a diverse range of national data sources from 2010 until 2023.

## 2 Methods

### 2.1 Data Sources

We collated publicly reported estimates of alcohol use and harms in Australia drawn from data sources with national or near‐national (e.g., majority of states/territories) coverage. Data sources and relevant measures for inclusion were identified by searching websites of national statistical and monitoring agencies (such as the AIHW and Australian Bureau of Statistics) and of universities engaged in alcohol monitoring (such as the National Drug and Alcohol Research Centre and the National Drug Research Institute) or through consultation with the research team who are experts in the field. Terminology used by reporting agencies has been adopted throughout. We aimed to obtain estimates capturing the last 15 years from 2010 until the most recent year of data available or years within this range. We have included key measures in the main text, with a range of supplementary measures reported throughout Supporting Information Material 1. Measure‐specific year ranges and definitions are in Table 1; further detail on data sources and especially their limitations is available in Table S1.

**TABLE 1:** Indicating definitions for each alcohol measure.

Measure type	Measurement	Definition	Population	Time period (frequency)	Data source	Disaggregation
Non‐survey alcohol consumption	Per capita consumption data	Total alcohol consumption per capita (in litres) computed from multiple data sources for each major drink type	15+	2010/11–2022/23 (yearly)	AIHW [22]	National only
	Per capita consumption via wastewater estimates	Alcohol consumption (in litres) from Australia‐wide wastewater analyses	All	2016–2024 (yearly)	NWDMP [23, 24, 25, 26, 27, 28, 29, 30, 31, 32, 33, 34, 35, 36, 37, 38, 39, 40, 41, 42, 43, 44, 45]	National or state‐based (estimations)
Survey alcohol consumption	High (weekly) risky drinker	Percentage of population consuming more than 10 standard drinks per week on average in the past 12 months	15+	2010–2022/23 (triennial)	NDSHS [46]	National or state‐based
	High (monthly) risky drinker	Percentage of population consuming more than 4 standard drinks on a single day at least once a month over the previous 12 months	15+	2010–2022/23 (triennial)	NDSHS [46]	National or state‐based
	Frequent risky drinker	Percentage of population who were current drinkers consuming at least 5 (if female) or 7 (if male) standard drinks on one occasion at least 2 or 3 times per month (by sex) in the previous 12 months	15+	2011–2021 (biennial)	HILDA [47]	National only
	Single‐occasion risky drinker	Percentage of population who exceeded guideline ‘no more than 4 standard drinks on a single occasion’ in the previous 12 months	18+	2012–2022 (triennial)	NHS [48]	National only
	Long‐term risky drinker	Percentage of population who exceeded guideline ‘no more than 2 standard drinks per day’ in the previous 12 months	18+	2012–2022 (triennial)	NHS [49]	National only
Alcohol‐related harms	Alcohol‐related deaths	Age‐standardised rate of death including both deaths directly attributable to alcohol and deaths where alcohol was listed as an associated cause	All	2010–2022 (yearly)	NMD [50]	National only
	Alcohol‐attributable deaths	Age‐standardised rate of all deaths where the underlying cause is either entirely or fractionally caused by alcohol according to calculated Australian‐specific alcohol‐attributable fractions	15+	2011–2022 (yearly)	NAIP [9]	National or state‐based
	Alcohol‐related hospitalisations	Age‐standardised rate of hospitalisations where the principal diagnosis was directly attributable to alcohol use	All	2011–2023 (yearly)	NIDIP [51]	National or state‐based
	Alcohol‐attributable hospitalisations	Age‐standardised rate of all hospitalisations based on either principal diagnoses or external causes relating to alcohol use according to calculated Australian‐specific alcohol‐attributable fractions	15+	2011–2022 (yearly)	NAIP [9]	National or state‐based
	Alcohol ambulance attendances	Rate of ambulance attendances with alcohol involvement, identified via paramedic clinical assessment, patient self‐report, information from third parties, and/or evidence at the scene	All	2015–2022 (yearly)	NASS [52]	National or state‐based (excluding WA and SA)
	Closed treatment episodes for alcohol	Rate of treatment episodes that were treatment for one's own use of alcohol where alcohol was listed as the principal drug of concern, determined due to being the main treatment type, reason for cessation, or source of referral	15+	2014/15–2022/23 (yearly)	AODTS NMDS [53]	National or state‐based
	Positive alcohol random breath tests	Proportion of random breath tests that reported a positive result for alcohol consumption	Drivers who were tested	2010–2022 (yearly)	BITRE [54]	National or state‐based (completeness varies by jurisdiction)

Abbreviations: AIHW, Australian Institute of Health and Welfare; AODTS NMDS, Alcohol and Other Drug Treatment Services National Minimum Dataset; BITRE, Bureau of Infrastructure and Transport Research Economics; HILDA, Household, Income and Labour Dynamics in Australia survey; NAIP, National Alcohol Indicators Project; NASS, National Ambulance Surveillance System; NDSHS, National Drug Strategy Household Survey; NHS, National Health Survey; NIDIP, National Illicit Drug Indicators Project; NMD, National Mortality Database; NWDMP, National Wastewater Drug Monitoring Program; SA, South Australia; WA, Western Australia.

#### 2.1.1 Non‐Survey Alcohol Consumption Measures

##### 2.1.1.1 Alcohol Available for Consumption

A measure of alcohol availability, specifically annual total per capita consumption (in litres) was obtained from the AIHW [22, 55]. This measure is the closest to a ‘gold standard’ measurement for overall population consumption given its use of multiple sources to quantify alcohol availability. However, it is limited due to its reliance on tax data and lack of availability of other sources, such as sales data, to inform estimates.

##### 2.1.1.2 National Wastewater Drug Monitoring Program

Estimates of per capita consumption from wastewater data were obtained from reporting by the Australian Criminal Intelligence Commission [56]. While wastewater estimates eliminate response‐related biases of other sources such as surveys, uncertainties regarding the reliability of estimates of consumption from waste sampling make it best used as a complementary measure [57].

#### 2.1.2 Survey Alcohol Consumption Measures

##### 2.1.2.1 National Drug Strategy Household Survey

Population estimates of risky alcohol consumption (according to the national alcohol intake guidelines [58]) were obtained from the National Drug Strategy Household Survey, a nationally representative triennial household survey [46]. Estimates of daily, risky, and current drinkers are noted in Supporting Information Material 1.

##### 2.1.2.2 Household, Income and Labour Dynamics in Australia (HILDA)

The HILDA is an annual household‐based panel survey [47]. A measure of frequent risky drinking was obtained, while measures of frequent, risky, and any current drinking are available in Supporting Information Material 1. All HILDA measures are reported by gender [47].

##### 2.1.2.3 National Health Survey

The National Health Survey is a nationally representative triennial survey, providing estimates of single‐occasion risky drinking and long‐term risky drinking [48].

While survey sources can be useful tools for monitoring population trends [14, 59], such measures are limited due to their reliance on self‐report methods, and depend on adequate response rates from the population (see Table S1 for ranges of response rates for each survey). Even well conducted surveys are prone to recall and social desirability bias as well as the exclusion of key population sub‐groups (e.g., people not in stable housing) from sampling frames [60]. Despite these limitations, researchers have shown that surveys can provide robust estimates of population trends, even while underestimating the actual level of consumption [59, 61].

#### 2.1.3 Alcohol‐Related Harms

##### 2.1.3.1 Mortality

Definitively assessing alcohol's contribution to deaths is complex, with various approaches taken to quantify the number of deaths that are either directly or indirectly related to alcohol consumption (see Table S1 for further information). We have focused on rates of alcohol‐related deaths and total alcohol‐attributable deaths here, while other indicators (wholly and partially alcohol‐attributable deaths, alcohol‐induced deaths and alcohol‐related injury deaths) are included in Supporting Information Material 1.

Alcohol‐related deaths capture deaths directly attributable to alcohol and where alcohol is listed as an associated cause. This measurement is constrained by changes in reporting practices over time, with some more recent data subject to further revision [62].

Alcohol‐attributable deaths estimate the total number of deaths related to alcohol by combining those where the underlying cause of death is wholly attributable to alcohol (e.g., alcohol toxicity) with a proportion of those that are partially (e.g., breast cancer) attributable to alcohol [9]. This data was preliminary at the time of data request, thus may underreport deaths where the cause of death was not confirmed until a coronial inquiry was completed [9, 63].

Deaths data are the least susceptible to biases due to service system practices or missing data, although they can be influenced by coding decisions around specific causes of death [64]. Deaths also capture only the most severe outcomes but are an essential measure capturing population harm from alcohol.

##### 2.1.3.2 Hospitalisations

Alcohol‐related hospitalisations capture hospitalisations where an alcohol‐related diagnosis code was identified in the principal diagnosis field [51]—in other words, the admission was for a condition definitively caused by alcohol (e.g., alcohol‐related liver disease).

Alcohol‐attributable hospitalisations report an estimate of the total number of hospitalisations attributable to alcohol by combining wholly attributable hospital admissions with attributable fractions of partially attributable admissions (e.g., some proportion of breast cancer admissions will be included). Supporting Information Material 1 contains these results split by wholly and partially attributable hospitalisations, with just the total estimated admissions included in the main document.

A measure of alcohol‐related injury hospitalisations has also been included in Supporting Information Material 1 [65].

##### 2.1.3.3 Ambulance Attendances

The National Ambulance Surveillance System provides data regarding ambulance attendances with identification of alcohol involvement from paramedic notes from 2015 to 2022 for all states and territories except for Western Australia and South Australia [52]. Annual all‐age rates were calculated based on averages of the quarterly rates reported by the National Ambulance Surveillance System [52].

##### 2.1.3.4 Treatment Episodes

The AIHW report yearly data regarding closed treatment episodes for alcohol use based on the Alcohol and Other Drug Treatment Services National Minimum Dataset [53]. Crude rates for 2014/2015–2022/2023 were manually calculated based on the number of treatment episodes for alcohol (where alcohol was the principal drug of concern for the client) and population numbers (aged 15+) sourced from the Australian Bureau of Statistics [66]. Treatment episodes provide a useful measure of population demand for alcohol treatment, but treatment rates are at least partly dependent on availability, so trends may not just measure the underlying rate of alcohol problems.

##### 2.1.3.5 Positive Random Breath Tests

Annual estimates of the proportion of random breath tests (RBT) positive for illegal blood alcohol concentration for 2010–2022 were calculated based on the number of positive RBTs and total number of RBTs reported by the Bureau of Infrastructure and Transport Research Economics, who source the data from multiple organisations [54]. Data completeness varies by year and jurisdictions, as detailed in the Road Safety Enforcement Data Dictionary [54] and the rate of positive tests will be dependent on enforcement decisions made by the police as well as the underlying rates of drink‐driving.

### 2.2 Data Analysis

Trends for all measures were assessed using Joinpoint version 5.4.0.0—a statistical software package developed by the United States National Cancer Institute [67]. Joinpoint regression enables the testing of changes in trends by determining the most parsimonious model with the optimal number of joinpoints (which is the time point at which the slope or rate of change changes). The software accepts a range of input data that can be used as dependent variables—including counts, rates and percent data—allowing the testing of trends for all alcohol‐related measures used in the current study. Joinpoint was chosen as it does not assume the presence of linear trends in the data, allowing for the testing of changes over time. It should be noted that the Joinpoint software is only able to assess for a single joinpoint with at least 7 points of data. However, this method was chosen to ensure a consistent analysis technique across all measures. For data with fewer than 7 points no joinpoints are assessed, and instead joinpoint simply fits a log‐linear model.

The minimum number of joinpoints was 0 (i.e., a straight line). We allowed the maximum number of joinpoints to be determined according to the algorithmic recommendations provided by the Joinpoint software—that is, a maximum of 2 joinpoints for trend data with 12–16 data points, a maximum of 1 joinpoint for data with 7–11 points and 0 joinpoints for data with 0–6 points [67], with the implications of this noted in the limitations. Where standard error estimates were provided (e.g., for alcohol consumption estimates from the National Drug Strategy Household Survey and National Health Survey), these estimates were used in analysis. For measures where standard error estimates were not available, we assumed homoscedasticity and selected the ‘constant variance’ option. The most parsimonious model, identified by the lowest Weighted Bayesian Information Criterion, is reported for each measure below.

The Joinpoint software reports both modelled trend values and annual percentage change (APC) values, calculated using a log‐linear model. The reported APCs therefore represent relative changes per year. Throughout our results, we assume a constant APC which is a standard assumption for such analyses [67, 68]. Modelled values were plotted alongside the observed values to generate visual trend graphs based on joinpoint analyses. The observed values and APC are also reported in Supporting Information tables for each variable. Data that were reported according to financial year (e.g., 2011–2012) were analysed according to the latest part of the year (e.g., 2012).

To summarise findings and facilitate easier comparisons, average APC were computed and reported for select studies that reported across a common time period (2012–2021). These average APC results are presented at the end of the results section. For all analyses, statistical significance was set at *α* = 0.05.

### 2.3 Ethical Approval and Registration

Ethical approval was not required for this study as all data were publicly available. This study was not pre‐registered.

## 3 Results

Figures 1, 2, 3, 4, 5, 6 indicate modelled joinpoint trends for each measure of alcohol consumption or harm, with Tables S2–S7 indicating the corresponding observed values and APC estimates for each measure.

**FIGURE 1:**
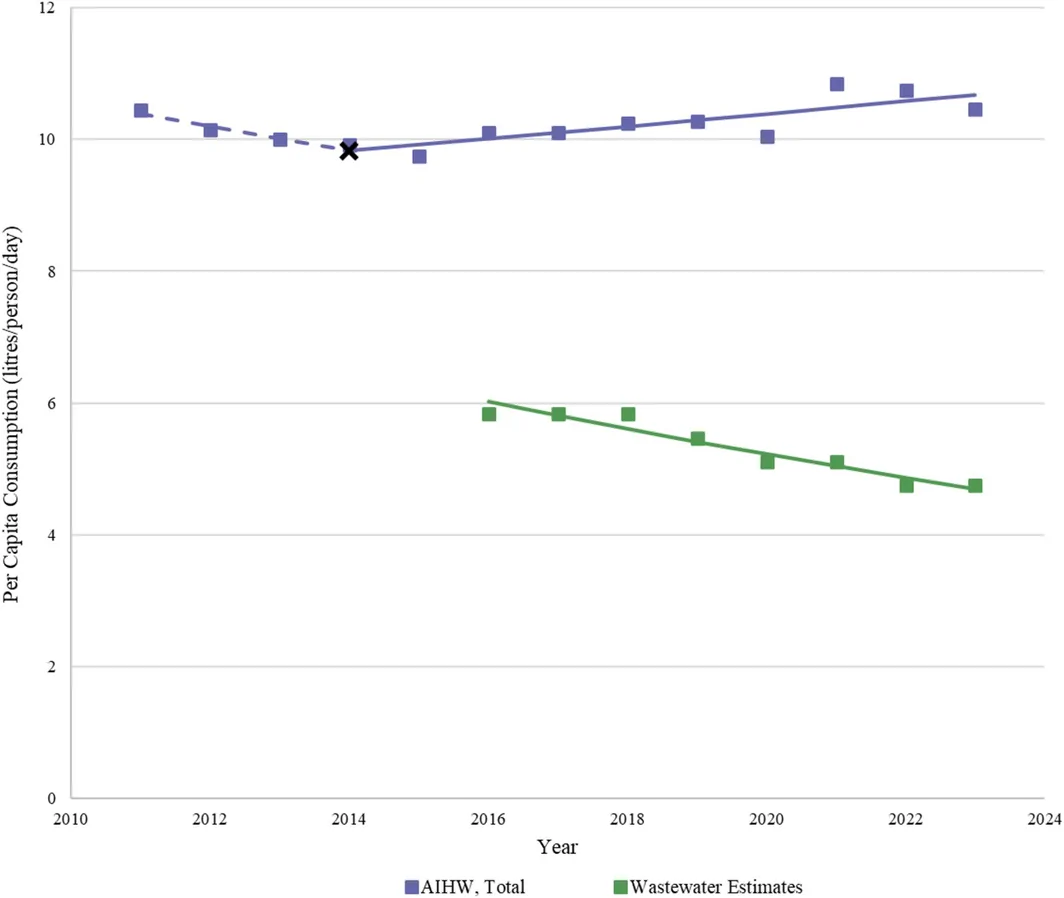
Modelled joinpoint trends indicating per capita alcohol consumption. *Note:* Observed values are indicated by scatter points and modelled trends indicated by line/dashed line. Statistically significant trends are indicated by solid lines and trends that are not statistically significant are indicated by dashed lines. A black cross indicates a joinpoint in the data. Australian Institute of Health and Welfare (AIHW) data are based on people 15+ years, and National Wastewater Drug Monitoring Program data are based on people of all ages.

**FIGURE 2:**
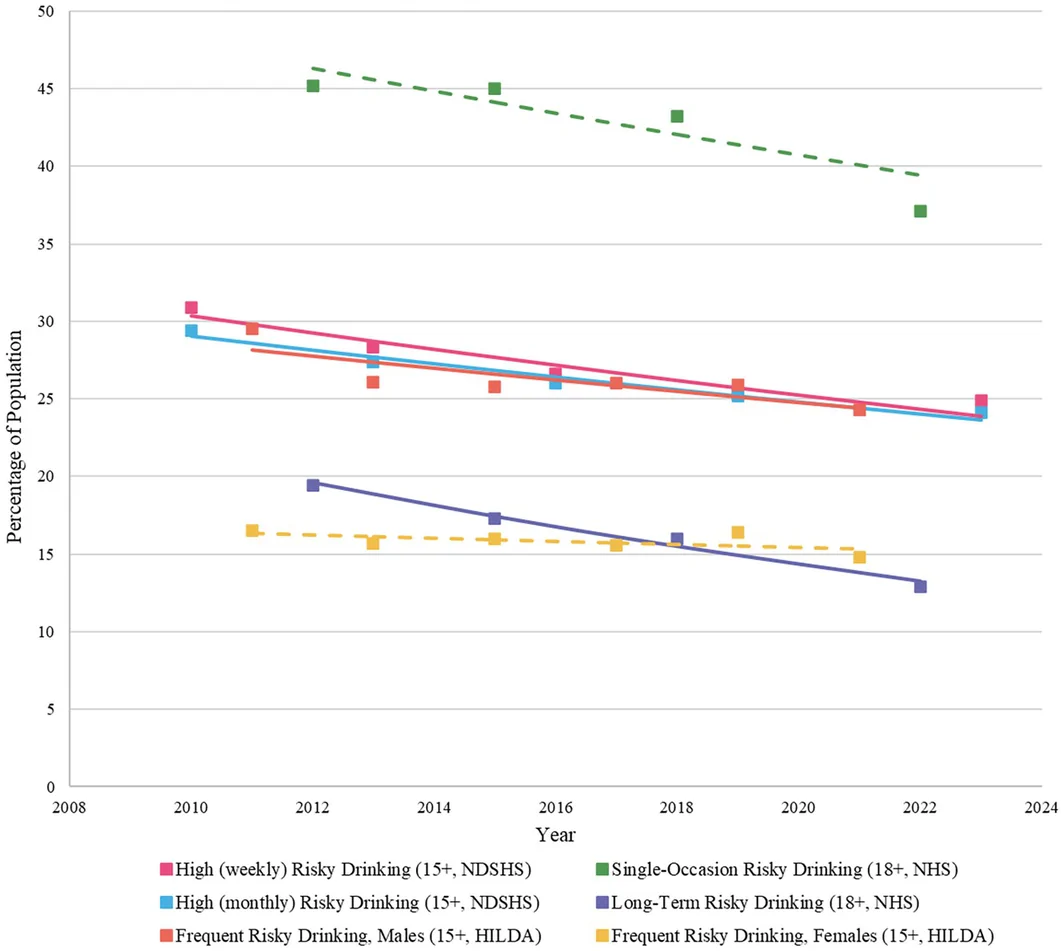
Modelled joinpoint trends indicating risky consumption measures. Definitions: High (weekly) risky drinker: more than 10 standard drinks per week on average in the past 12 months. High (monthly) risky drinker: more than four standard drinks on a single day at least once a month over the previous 12 months. Single‐occasion risky drinking: Exceeded guideline ‘no more than 4 standard drinks on a single occasion’ in the previous 12 months. Long‐term risky drinking: Exceeded guideline ‘no more than 2 standard drinks per day’ in the previous 12 months. Frequent risky drinker: Of current drinkers, consume at least 5 (if female) or 7 (if male) standard drinks on one occasion at least two or three times per month. *Note:* Observed values are indicated by scatter points and modelled trends indicated by line/dashed line. Statistically significant trends are indicated by solid lines and trends that are not statistically significant are indicated by dashed lines. Abbreviations: HILDA, Household, Income and Labour Dynamics in Australia Survey; NDSHS, National Drug Strategy Household Survey; NHS, National Health Survey.

**FIGURE 3:**
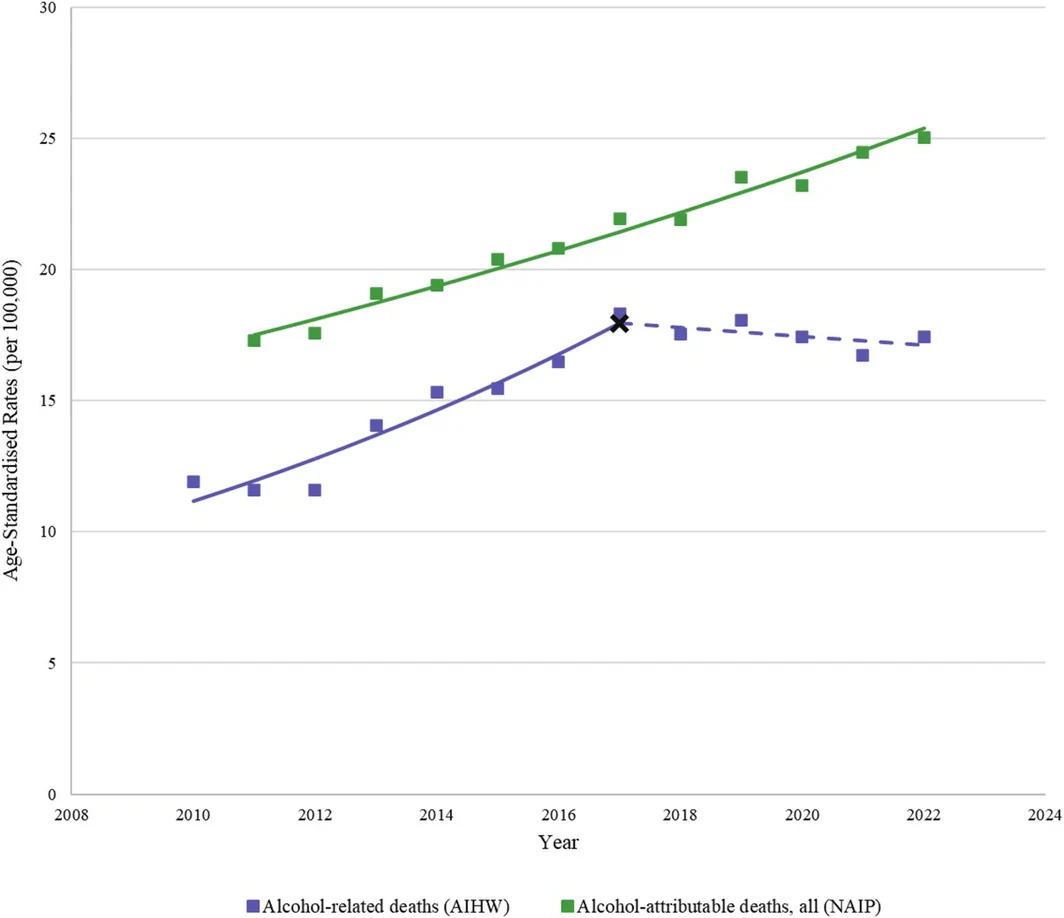
Modelled joinpoint trends indicating deaths related to alcohol. Definitions: Alcohol‐related deaths: Including both deaths directly attributable to alcohol and deaths where alcohol was listed as an associated cause. Alcohol‐attributable deaths: deaths where the underlying cause is either entirely or fractionally caused by alcohol according to calculated Australian‐specific alcohol‐attributable. *Note:* Observed values are indicated by scatter points, and modelled trends indicated by line/dashed line. Statistically significant trends are indicated by solid lines and trends that are not statistically significant are indicated by dashed lines. A black cross indicates a joinpoint in the data. Abbreviations: AIHW, Australian Institute of Health and Welfare; NAIP, National Alcohol Indicators Project.

**FIGURE 4:**
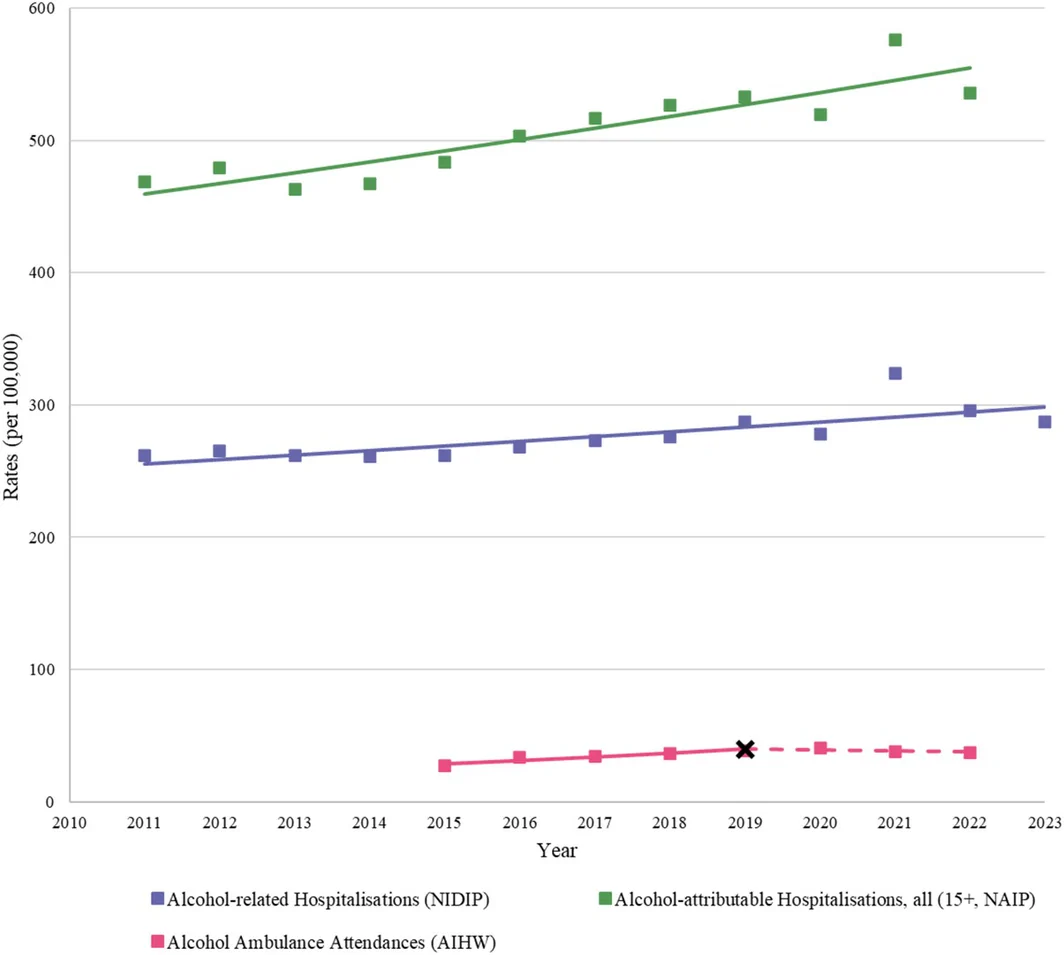
Modelled joinpoint trends indicating hospitalisations and related harms due to alcohol. Definitions: Alcohol‐related hospitalisations: hospitalisations where the principal diagnosis was directly attributable to alcohol use. Alcohol ambulance attendances: Ambulance attendances with alcohol involvement, identified via paramedic clinical assessment, patient self‐report, information from third parties and/or evidence at the scene. Alcohol‐attributable hospitalisations: hospitalisations based on either primary diagnoses or external causes relating to alcohol use according to calculated Australian‐specific alcohol‐attributable fractions. *Note:* It should be noted that the ambulance attendance values do not include Western Australia or South Australia, so is not wholly national data. Observed values are indicated by scatter points and modelled trends indicated by line/dashed line. Statistically significant trends are indicated by solid lines and trends that are not statistically significant are indicated by dashed lines. A black cross indicates a joinpoint in the data. Abbreviations: AIHW, Australian Institute of Health and Welfare; NAIP, National Alcohol Indicators Project; NIDIP, National Illicit Drug Indicators Project.

**FIGURE 5:**
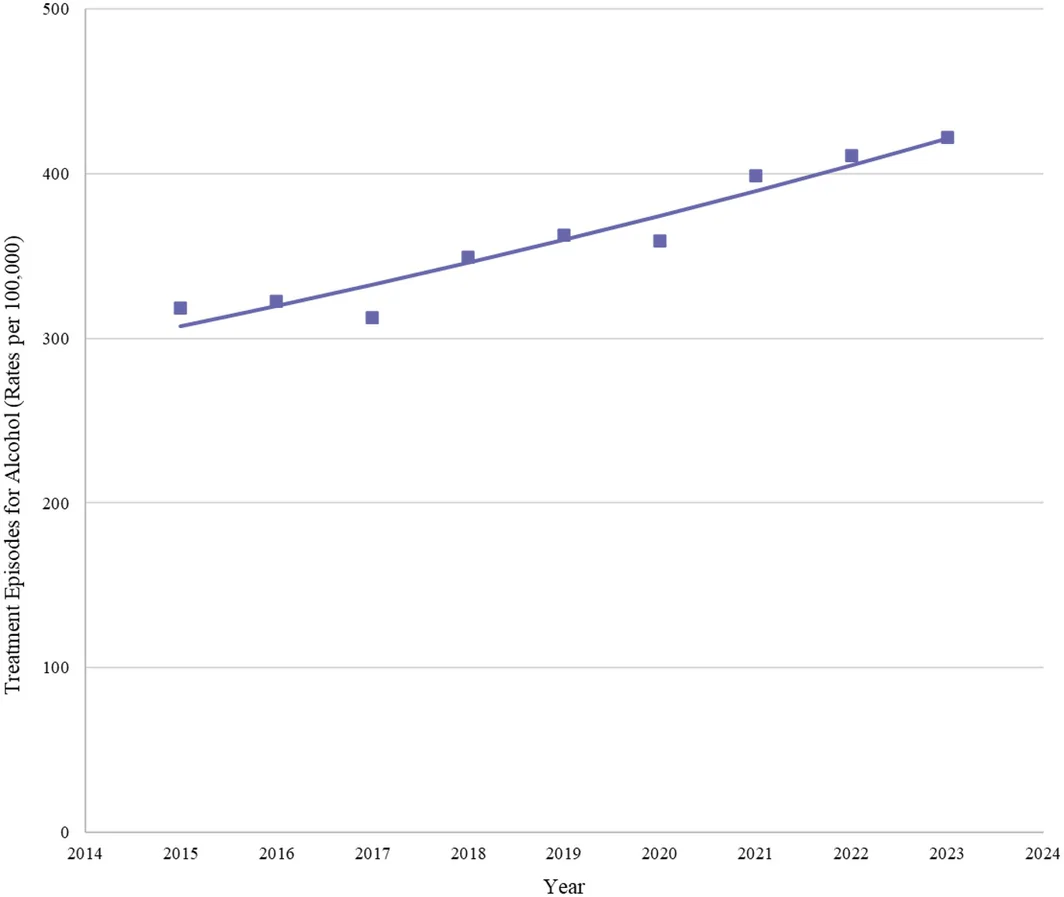
Modelled joinpoint trends indicating closed treatment episodes for alcohol. Definition of closed treatment episode: treatment for one's own use where alcohol was listed as the principal drug of concern, determined due to being the main treatment type, reason for cessation, or source of referral. *Note:* Observed values are indicated by scatter points, and modelled trends indicated by line/dashed line. Statistically significant trends are indicated by solid lines and trends that are not statistically significant are indicated by dashed lines.

**FIGURE 6:**
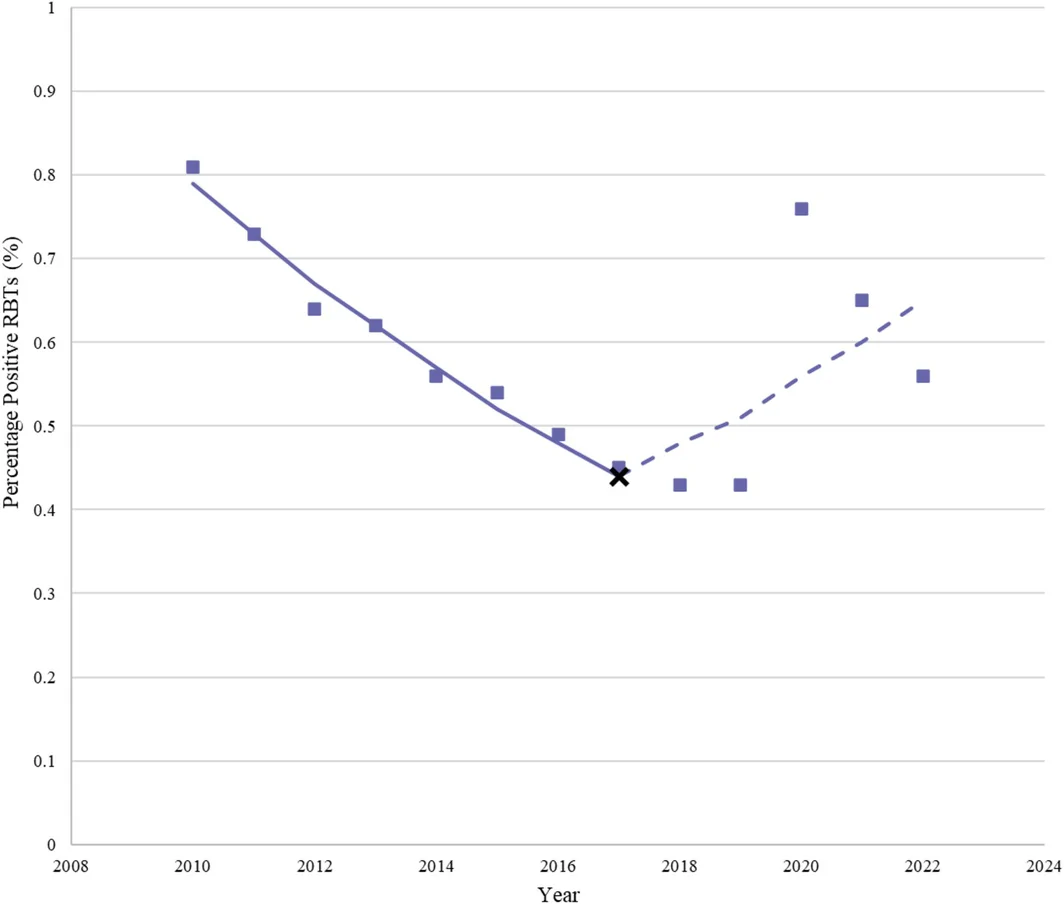
Modelled joinpoint trends indicating the proportion (%) of positive random breath tests for alcohol. *Note:* Observed values are indicated by scatter points, and modelled trends indicated by line/dashed line. Statistically significant trends are indicated by solid lines and trends that are not statistically significant are indicated by dashed lines. A black cross indicates a joinpoint in the data. RBT, random breath tests (for alcohol).

### 3.1 Non‐Survey Consumption Measures

Visualisations of the joinpoint analyses for per capita consumption are in Figure 1, with observed values and APC estimates found in Table S2. Per capita consumption according to the AIHW was relatively stable between 2011 and 2014, before reporting a significant increase of 0.92% per year over a 9‐year period from 2014 to 2023. Conversely, according to NWDMP wastewater trends, per capita consumption declined by 3.46% per year from 2016 to 2023.

### 3.2 Survey Alcohol Consumption Measures

Results for survey data capturing trends in risky drinking are reported in Figure 2 and Table S3. There were no joinpoints in trends for survey consumption measures due to the limited number of data points.

Risky consumption has mostly declined from 2010 onwards. This was by a sizeable amount for most measures, such as an annual 3.84% decline in long‐term risky drinking from 2012 to 2022 (from the National Health Survey) and a 1.83% decline in high (weekly) risky drinking from 2010 to 2023 (from NDSHS), per year. HILDA survey results indicate significant declines only for male frequent risky drinkers (annual decline of 1.40% between 2011 and 2021), while female frequent risky drinkers indicated non‐significant trends.

Results for supplementary measures can be found in Supporting Information Material 1 (Part 1), with trends in other measures (e.g., any drinking, frequent drinking) broadly in line with the risky drinking measures here.

### 3.3 Alcohol‐Related Harm Measures

Mortality data results are reported in Figure 3 and Table S4. Alcohol‐related deaths from the AIHW significantly increased 7.03% per year from 2010 to 2017, before a period of non‐significant decline of 0.96% from 2018 to 2022. Total alcohol‐attributable deaths from the NAIP saw an increase of 3.43% per year over a similar time period from 2011 to 2022.

Results regarding hospitalisations related to alcohol (Figure 4, Table S5) have generally significantly increased—both in alcohol‐related hospitalisations from NIDIP, which increased annually by 1.30% from 2011 to 2023, and in total alcohol‐attributable hospitalisations from NAIP, where rates increased by 1.72% per year from 2011 to 2022. Ambulance attendances due to alcohol significantly increased by 8.57% per year over a four‐year period until 2019, with non‐significant decreasing trends after this time.

Results for supplementary measures relating to mortality and hospitalisations can be found in Supporting Information Material 1 (Part 2) and also show generally increasing trends.

### 3.4 Treatment

Treatment data results are reported in Figure 5 and Table S6. Treatment episodes for alcohol have increased by 4.03% per year over the entire period 2015–2023.

### 3.5 RBTs

Data regarding the proportion of positive RBTs for alcohol (Figure 6, Table S7) indicated a significant decline of 8.12% per year reported from 2010 to 2017, with a non‐significant increasing trend of 8.25% per year after this time point.

### 3.6 Summary

By way of summary, Table 2 indicates APC for all included measures using a colour‐coded system—APC for supplementary measures can be found in Supporting Information Material 1 (Part 3). Most consumption variables show declining trends over time (as indicated by the blue coloured cells), while most alcohol‐related harm variables show increasing trends (indicated by the red coloured cells). The contrast in trends between per capita consumption and wastewater estimates is especially evident in this table, as are the consistently declining trends in survey measures of consumption. Some alcohol‐related harms, such as alcohol‐related deaths and ambulance attendances, stand out strongly with dramatic increases.

**TABLE 2:** Annual percentage change for each measure of alcohol‐related consumption and harm per year, according to Joinpoint analyses.

Measure	2010	2011	2012	2013	2014	2015	2016	2017	2018	2019	2020	2021	2022	2023
***Non‐survey consumption measures***														
Per capita consumption *(L) Total, AIHW* ^a^		−1.86	−1.86	−1.86	**0.92**	**0.92**	**0.92**	**0.92**	**0.92**	**0.92**	**0.92**	**0.92**	**0.92**	**0.92**
Wastewater ethanol estimates *(L/1000 person/day), NWDMP* ^b^							**−3.46**	**−3.46**	**−3.46**	**−3.46**	**−3.46**	**−3.46**	**−3.46**	**−3.46**
***Survey consumption measures***														
High (weekly) risky drinkers *15+ (%), NDSHS* ^c^	**−1.83**	**−1.83**	**−1.83**	**−1.83**	**−1.83**	**−1.83**	**−1.83**	**−1.83**	**−1.83**	**−1.83**	**−1.83**	**−1.83**	**−1.83**	**−1.83**
High (monthly) risky drinkers *15+ (%), NDSHS* ^c^	**−1.57**	**−1.57**	**−1.57**	**−1.57**	**−1.57**	**−1.57**	**−1.57**	**−1.57**	**−1.57**	**−1.57**	**−1.57**	**1.57**	**−1.57**	**−1.57**
Long‐term risky drinkers *18+ (%), NHS* ^c^			**−3.84**	**−3.84**	**−3.84**	**−3.84**	**−3.84**	**−3.84**	**−3.84**	**−3.84**	**−3.84**	**−3.84**	**−3.84**	
Single‐occasion risky drinkers *18+ (%), NHS* ^c^			−1.59	−1.59	−1.59	−1.59	−1.59	−1.59	−1.59	−1.59	−1.59	−1.59	−1.59	
Frequent risky drinker *Males 15+ (%), HILDA* ^c^		**−1.40**	**−1.40**	**−1.40**	**−1.40**	**−1.40**	**−1.40**	**−1.40**	**−1.40**	**−1.40**	**−1.40**	**−1.40**		
Frequent risky drinker *Females 15+ (%), HILDA* ^c^		−0.62	−0.62	−0.62	−0.62	−0.62	−0.62	−0.62	−0.62	−0.62	−0.62	−0.62		
***Alcohol‐related harm measures***														
Alcohol‐related deaths *entire pop. (per 100,000), AIHW* ^a^	**7.03**	**7.03**	**7.03**	**7.03**	**7.03**	**7.03**	**7.03**	−0.96	−0.96	−0.96	−0.96	−0.96	−0.96	
Alcohol‐attributable deaths *15+ (per 100,000)—All, NAIP* ^a^		**3.43**	**3.43**	**3.43**	**3.43**	**3.43**	**3.43**	**3.43**	**3.43**	**3.43**	**3.43**	**3.43**	**3.43**	
Alohol‐related hospitalisations *entire pop. (per 100,000), NIDIP* ^a^		**1.30**	**1.30**	**1.30**	**1.30**	**1.30**	**1.30**	**1.30**	**1.30**	**1.30**	**1.30**	**1.30**	**1.30**	**1.30**
Alcohol‐attributable hospitalisations *15+ (per 100,000)—All, NAIP* ^a^		**1.72**	**1.72**	**1.72**	**1.72**	**1.72**	**1.72**	**1.72**	**1.72**	**1.72**	**1.72**	**1.72**	**1.72**	
Alcohol ambulance attendances *entire pop. (per 100,000), AIHW* ^b^						**8.57**	**8.57**	**8.57**	**8.57**	−2.06	−2.06	−2.06	−2.06	
Closed treatment episodes for alcohol *15+ (per 100,000), AIHW* ^b^					**4.03**	**4.03**	**4.03**	**4.03**	**4.03**	**4.03**	**4.03**	**4.03**	**4.03**	**4.03**
Positive RBTs for alcohol *Driving population (%), BITRE* ^a^	**−8.12**	**−8.12**	**−8.12**	**−8.12**	**−8.12**	**−8.12**	**−8.12**	8.25	8.25	8.25	8.25	8.25	8.25	

*Note:* Bolded black text indicates significant trend at *p* ≤ 0.05, non‐bolded text indicates non‐significant trend. Lined cell border indicates Joinpoint. Blue cells indicate decreasing trends, red cells indicated increasing trends. Strength of shading indicates greater trend (i.e., annual percentage change of 0–5 are lightest, 5–10 are medium and 15+ darkest shades). Some data sources (such as NDSHS) are not annual data (see Table 1 for further information), however, Joinpoint provides annual values indicating percentage change.

Abbreviations: AIHW, Australian Institute of Health and Welfare; BITRE, Bureau of Infrastructure and Transport Research Economics; HILDA, Household, Income and Labour Dynamics in Australia survey; NAIP, National Alcohol Indicators Project; NDSHS, National Drug Strategy Household Survey; NHS, National Health Survey; NIDIP, National Illicit Drug Indicators Project; NWDMP, National Wastewater Drug Monitoring Program; RBT, random breath test.

^a^ Measure with 12–16 data points and a maximum of two joinpoints tested.

^b^ Measure with 7–11 data points and a maximum of 1 joinpoint tested.

^c^ Measure with 0–6 data points and 0 joinpoints tested.

This is further reinforced in Table 3 (and Supporting Information Material 1 (Part 3)), indicating the average APC values for most measures. Measures were included in this table if they shared a common time period, being 2012–2021. Over the same time period, all survey measures of consumption showing significant trends indicate an average APC decline between 1.40% and 3.83% per year, while all significant harm trends showed increases up to a maximum of 3.43% per year for alcohol‐attributable deaths.

**TABLE 3:** Table indicating average annual percentage change (APC) for measures with a time period spanning 2012–2021.

Measure type	Measurement	Average APC	Confidence intervals
Non‐survey alcohol consumption	Per capita consumption data (total, AIHW)	0.30	[−0.45, 1.06]
Survey alcohol consumption	High (weekly) risky drinker (NDSHS)	−1.83*	[−2.75, −0.90]
	High (monthly) risky drinker (NDSHS)	−1.57*	[−2.12, −1.02]
	Frequent risky drinker (HILDA)		
	Males	−1.40*	[−2.69, −0.09]
	Females	−0.62	[−1.80, 0.57]
	Single‐occasion risky drinker (NHS)	−1.59	[−4.38, 1.27]
	Long‐term risky drinker (NHS)	−3.84*	[−5.65, −1.99]
Alcohol‐related harms	Alcohol‐related deaths (AIHW)	3.40*	[1.65, 5.19]
	Alcohol‐attributable deaths (all, NAIP)	3.43*	[3.06, 3.80]
	Alcohol‐related hospitalisations (NIDIP)	1.30*	[0.66, 1.95]
	Alcohol‐attributable hospitalisations (all, NAIP)	1.72*	[1.15, 2.29]
	Positive alcohol random breath tests (BITRE)	−1.17	[−6.02, 3.92]

*Note:* The following measures are not included in this table, as they did not have data for the entire 2012–2021 period for comparison: wastewater estimates, alcohol ambulance attendances (AIHW), and closed treatment episodes for alcohol (AIHW).

Abbreviations: AIHW, Australian Institute of Health and Welfare; BITRE, Bureau of Infrastructure and Transport Research Economics; HILDA, Household, Income and Labour Dynamics in Australia survey; NAIP, National Alcohol Indicators Project; NDSHS, National Drug Strategy Household Survey; NHS, National Health Survey; NIDIP, National Illicit Drug Indicators Project.

* Significant average APC at *p* ≤ 0.05.

## 4 Discussion

This study presented publicly accessible data across a range of sources assessing long‐term trends in alcohol consumption and harm from 2010. Results indicated that survey‐based measures of both current drinking and risky drinking, as well as wastewater estimates of per capita alcohol consumption, have largely declined for the Australian population over the last 15 years. However, the AIHW's per‐capita consumption measure, and measures of alcohol‐related harms including deaths, hospitalisations, ambulance attendances and treatment episodes, have mostly increased over the same time period.

While literature comparing trends in both consumption and harms is scarce and mostly based in the 2000s, similarities between these studies and the current results are present. For example, trends in Victoria from 2000 to 2008 comparing survey measures of risky drinking and alcohol‐related harms using health systems data, police data and vehicle crash data indicated sharp increases in alcohol‐related harms over the period, despite stable trends in risky drinking [10]. In a similar study focused specifically on young people aged 10–24 in Victoria, stable trends in risky drinking were overlaid with a dramatic increase in harms, especially acute intoxication‐related harms, until the mid‐2000s [69]. A study comparing trends Australia‐wide showed relatively stable per capita consumption despite increases in alcohol‐attributable harms, concluding that health harms attributable to alcohol increased over the period 2001–2010 [18].

Per capita consumption (our most robust measure of drinking trends) reported by the AIHW had increased moderately alongside harm rates, at least since 2014. This is more in line with the well‐established evidence that harm rates tend to move along with population levels of drinking [70, 71]. However, the average increase in per capita consumption over the study period was 0.3% per year, compared with increases in mortality of between 1.6% and 3.4% per year, so rates of harms are still increasing more quickly than overall consumption. These trends indicate patterns consistent with polarisation of drinking, with heavy drinkers increasing their risk of harm even while population levels of drinking remain relatively stable. Early analyses of UK data suggest that this has been partly responsible for the sharp increases in alcohol‐related mortality observed there during the pandemic years [72]. More research into trends in drinking among heavy drinkers and vulnerable populations is critical to better understand what is driving the trends we report here. Notably, despite significant increases for all other harm measures, a strong decline in RBTs from 2010 to 2017 was reported. However, the collection of such data relies on factors such as police presence and funding, with RBT data noted to have a range of completeness issues and inconsistencies in definition and data collection practices [54]. Therefore, this measure of harm is not considered as important as others, such as deaths data, which are routinely collected for the entire population.

The divergence between our survey‐based estimates of consumption which generally show clear declines and the per‐capita measure from the AIHW raises important concerns about the ongoing validity of population surveys when measuring trends in consumption. Previous work showed that estimates from surveys had moved broadly in concert with per‐capita measures [14], but that may no longer be the case. The striking declining trend in wastewater data adds another complication to interpretation here, but the uncertainties about the reliability of trend estimates from wastewater are still substantial [57]. The AIHW measure of alcohol available for consumption [22] is generally considered the ‘gold standard’ measurement of population consumption in Australia, but it relies on complex estimates compiling tax, import and survey data (especially for wine, where excise taxes are not charged making estimation more difficult). Robust national estimates of alcohol sales at the wholesale or retail level would provide the best possible estimates of population drinking levels.

### 4.1 Limitations

Limitations in the current study are reflected in the limitations of the data sources used (see Table S1). Some data had specific limitations—for example, the alcohol ambulance attendance data are not truly national data, while per capita consumption wastewater data were based on author‐generated estimates only. Survey data that monitors population‐level alcohol consumption are subject to a range of biases, and while still useful to track broader trends in the population [14, 59], are known to underestimate population alcohol consumption and have increasing issues regarding representativeness and sample size [13, 73, 74]. We also acknowledge the absence of sources that are not currently publicly available, such as national emergency department data or alcohol sales data. Finally, it should be noted that some measures had only minimal numbers of data points available for analysis, and the Joinpoint software is only able to assess for a single joinpoint with at least 7 points of data, or two joinpoints at 11 or more data points [67]. All survey consumption measures included 6 or less points of data, while non‐survey consumption and alcohol‐related harm measures included 7 or more points of data. While the overarching trends are still relevant for interpretation, due to the lack of data points in some sources, it remains unclear whether this constraint obscured any joinpoints that may otherwise have been present in the data.

While the diverging trends in alcohol consumption and related harms are clear on a population level, the current study is limited in its focus only on such broader population‐level statistics. Future work would be useful to disaggregate these trends by subsections of the population to understand which groups are contributing to the rise in alcohol‐related harms, compared to the decline in overall consumption. For example, although a wealth of evidence has documented the decline in alcohol consumption by adolescents and young adults in Australia since the 2000s [14, 75, 76], there has been much less evidence tracking trends in alcohol‐related harms for this population. Further work synthesising consumption and harm data for these subpopulations in Australia would be useful in generating a deeper understanding of the key population drivers of diverging consumption and harm trends.

Despite these limitations, the key strength of this study is its synthesis of multiple indicators to provide a robust understanding of alcohol consumption and related harms among the Australian general population. This multi‐dataset approach is valuable as it minimises the biases inherent in any one dataset [11], analysing all data sources together to provide a cohesive overview of trends over time, and allows results to be collated and interpreted together. This population‐level overview of Australian trends in consumption and harms would be useful to compare with other countries. However, for such international generalisability to be feasible, countries would require data availability similar to or beyond (in the case of alcohol sales data) what we have access to within Australia to provide a comprehensive estimate of trends for comparison.

## 5 Conclusions

Over the past 15 years, trends in alcohol consumption have declined for the Australian population, while alcohol‐related harms have increased over the same period. Notably, these trends were consistent for multiple types of consumption (i.e., risky and non‐risky drinking) and harms (i.e., hospitalisations and deaths), although aggregated consumption measures based on tax and import data did not show a decline. The synthesis of multiple sources of data strengthens the results of this study and lessens the impact of biases inherent in the individual data sources, highlighting the need a comprehensive approach to synthesising key measures tracking alcohol‐related consumption and harm. Further research replicating these methods in discrete subpopulations would be useful in understanding these emerging divergent trends in alcohol consumption and harm. These findings are an important step in the long‐term monitoring of national trends in alcohol consumption and related harm.

## Author Contributions

Each author certifies that their contribution to this work meets the standards of the International Committee of Medical Journal Editors.

## Funding

The National Drug and Alcohol Research Centre is funded by the Australian Government Department of Health, Disability and Ageing under the Drug and Alcohol Program. A.P. is supported by a National Health and Medical Research Council Investigator Fellowship (#1174630). M.L. is supported by the Australian Research Council (FT210100656).

## Conflicts of Interest

The authors declare no conflicts of interest.

## Supporting information


**Table S1:** Information on data sources and limitations.
**Table S2:** Observed values, annual percentage change (APC) and confidence intervals (CI) indicating trends for per capita consumption based on alcohol available for consumption data (sourced from the AIHW) and wastewater estimates (sourced from the NWDMP).
**Table S3:** Observed values, annual percentage change (APC) and confidence intervals (CI) indicating trends in risky drinking (%) among the population, sourced from the NDSHS, NHS and HILDA survey.
**Table S4:** Observed values, annual percentage change (APC) and confidence intervals (CI) indicating trends in deaths related to alcohol (age‐standardised rates per 100,000 population), sourced from the AIHW and NAIP.31.
**Table S5:** Observed values, annual percentage change (APC) and confidence intervals (CI) indicating trends in alcohol‐related hospitalisations (per 100,000 population), sourced from the AIHW and NAIP.
**Table S6:** Observed values, annual percentage change (APC) and confidence intervals (CI) indicating trends in closed treatment episodes for alcohol (per 100,000 population), sourced from the AIHW.
**Table S7:** Observed values, annual percentage change (APC) and confidence intervals (CI) indicating trends in the proportion (%) of positive random breath tests for alcohol, sourced from BITRE.

## Data Availability

Research data are not shared.
