# Supplementary material for: Trends in Australian Alcohol Consumption and Related Harms, 2010–2023
Source: Drug Alcohol Rev. 2026 Oct 19;45(7):e70261. doi: 10.1111/dar.70261 (PMC13678405; doi:10.1111/dar.70261)
Supplement: Supplementary file 1 [file DAR-45-0-s001.docx]

# Supporting Information

**Table of Contents**

[Supporting Information	1](#_Toc239788063)

[Supplementary Material 1. Methods and results for all supplementary data sources.	2](#_Toc239788064)

[Supplementary Methods:	2](#_Toc239788065)

[Supplementary Results:	5](#_Toc239788066)

[Table S1. Information on data sources & limitations	15](#_Toc239788067)

[Table S2. Observed values, annual percentage change (APC) and confidence intervals (CI) indicating trends for per capita consumption based on alcohol available for consumption data (sourced from the AIHW) and wastewater estimates (sourced from the NWDMP).	28](#_Toc239788068)

[Table S3. Observed values, annual percentage change (APC) and confidence intervals (CI) indicating trends in risky drinking (%) among the population, sourced from the NDSHS, NHS and HILDA survey (continued on next page)	29](#_Toc239788069)

[Supplementary Table 4. Observed values, annual percentage change (APC) and confidence intervals (CI) indicating trends in deaths related to alcohol (age-standardised rates per 100,000 population), sourced from the AIHW and NAIP.	31](#_Toc239788070)

[Table S5. Observed values, annual percentage change (APC) and confidence intervals (CI) indicating trends in alcohol-related hospitalisations (per 100,000 population), sourced from the AIHW and NAIP.	32](#_Toc239788071)

[Table S6. Observed values, annual percentage change (APC) and confidence intervals (CI) indicating trends in closed treatment episodes for alcohol (per 100,000 population), sourced from the AIHW.	33](#_Toc239788072)

[Table S7. Observed values, annual percentage change (APC) and confidence intervals (CI) indicating trends in the proportion (%) of positive Random Breath Tests for alcohol, sourced from BITRE	34](#_Toc239788073)

## Supplementary Material 1. Methods and results for all supplementary data sources.

### Supplementary Methods:

Definitions and further information regarding measures not included in the main manuscript can be found below in Table 1. Please see Supplementary Table 1 (next section below) for further detail on these data sources and their limitations.

**Table A.** Table indicating definitions for each alcohol measure not included in the main manuscript.

| Measure type | Measurement | Definition | Population | Time period (*Frequency*) | Data source |
| --- | --- | --- | --- | --- | --- |
| Survey alcohol consumption | Daily drinker | Percentage of population consuming any alcohol daily | 15+ | 2010-2022/23  *(triennial)* | NDSHS(1) |
|  | Risky drinker | Percentage of population consuming more than 10 standard drinks per week or more than 4 standard drinks on a single day at least once a month on average in the past 12 months | 15+ | 2010-2022/23  *(triennial)* | NDSHS(1) |
|  | Current drinker | Percentage of population who consumed alcohol in the past 12 months | 15+ | 2010-2022/23  *(triennial)* | NDSHS(1) |
|  | Frequent drinker | Percentage of population drinking on 5 or more days per week (by sex) in the previous 12 months | 15+ | 2011-2021 *(biennial)* | HILDA(2) |
|  | Risky drinker | Percentage of population who were current drinkers and usually consume 5 or more standard drinks on one occasion (by sex) in the previous 12 months | 15+ | 2011-2021 *(biennial)* | HILDA(2) |
|  | Current drinker | Percentage of population who reported ever drinking alcohol (by sex) in the previous 12 months | 15+ | 2011-2021 *(biennial)* | HILDA(2) |
| Alcohol-related harms | Alcohol-attributable deaths | Age-standardised rate of deaths where the underlying cause is either entirely or fractionally caused by alcohol according to calculated Australian-specific alcohol-attributable fractions (including wholly or partially) | 15+ | 2011-2022  *(yearly)* | NAIP(3) |
|  | Alcohol-induced deaths | Age-standardised rate of death where the underlying cause was directly attributable to alcohol use | All | 2010-2023  *(yearly)* | NIDIP(4) |
|  | Alcohol-related injury deaths | Age-standardised rate of deaths with either a principal diagnosis of injury and additional in-scope diagnosis relating to alcohol, or external cause of injury relating to alcohol | All | 2011-2020  *(yearly)* | NMD(5) |
|  | Alcohol-attributable hospitalisations | Age-standardised rate of hospitalisations based on either principal diagnoses or external causes relating to alcohol use according to calculated Australian-specific alcohol-attributable fractions (including wholly or partially) | 15+ | 2011-2022  *(yearly)* | NAIP(3) |
|  | Alcohol-related injury hospitalisations | Rate of hospitalisations with either a principal diagnosis of injury and additional in-scope diagnosis relating to alcohol, or external cause of injury relating to alcohol | All | 2011-2017  *(yearly)* | NHMD(5) |

Abbreviations: HILDA, Household, Income and Labour Dynamics in Australia survey; NAIP, National Alcohol Indicators Project; NDSHS, National Drug Strategy Household Survey; NHMD, National Hospital Morbidity Database; NHS, National Health Survey.

**References**

1.	Australian Institute of Health and Welfare. National Drug Strategy Household Survey 2022–2023. AIHW, Australian Government; 2024. Available from: <https://www.aihw.gov.au/reports/illicit-use-of-drugs/national-drug-strategy-household-survey/contents/about>

2.	Wilkins R, Vera-Toscano E, Botha F. The Household, Income and Labour Dynamics in Australia Survey: Selected Findings from Waves 1 to 21. Melbourne Institute: Applied Economic & Social Research, the University of Melbourne; 2024. Available from: <https://melbourneinstitute.unimelb.edu.au/hilda/publications/hilda-statistical-reports>

3.	Gilmore W, Lensvelt E, Jones P, Dorocicz J, Sherk A, Churchill S, et al. *Australian alcohol-attributable harm visualisation tool version 2.1.* [Internet]. National Drug Research Institute, Curtin University and Canadian Institute for Substance Use Research, University of Victoria, 2023. www.alcoholharmtool.info.

4.	Chrzanowska A MN, Sutherland R, Degenhardt L, Peacock A. Trends in overdose and other drug-induced deaths in Australia, 2004-2023. Sydney: National Drug and Alcohol Research Centre, UNSW; 2025. Available from: <https://doi.org/10.26190/unsworks/31245>

5.	Australian Institute of Health and Welfare. Alcohol-related injury: hospitalisations and deaths, 2019–20. AIHW, Australian Government; 2023. Available from: <https://www.aihw.gov.au/reports/injury/alcohol-related-injuries-2019-20/contents/about>

### Supplementary Results:

#### Part 1. Survey Alcohol Consumption Measures

Figure indicating modelled Joinpoint trends reporting current consumption measures

Figure indicating modelled Joinpoint trends reporting additional risky consumption measures

*Definitions: Risky drinker (NDSHS): More than 10 standard drinks per week or more than 4 standard drinks on a single day at least once a month on average. Risky drinker (HILDA): Of current drinkers, usually consume 5 or more standard drinks on one occasion. Daily drinker: Consumed alcohol daily. Frequent drinker: drinking on 5 or more days per week.*
Note: Observed values are indicated by scatter points, and modelled trends indicated by line/dashed line. Statistically significant trends are indicated by solid lines and trends that are not statistically significant are indicated by dashed lines.

*Definitions: Current drinker: Consumption of alcohol in the previous 12 months.* Note: Observed values are indicated by scatter points, and modelled trends indicated by line/dashed line. Statistically significant trends are indicated by solid lines and trends that are not statistically significant are indicated by dashed lines.

**Table B.** Observed values, Annual Percentage Change (APC), and confidence intervals (CI) indicating trends in drinking (%) among the population, sourced from the NDSHS and HILDA survey (continued on next pages)

| Year | Daily drinkers, *15+ (NDSHS)* | APC | 95% CIs | Risky drinkers,  *15+ (NDSHS)* | APC | 95% CIs | Current drinkers,  *15+ (NDSHS* | APC | 95% CIs |
| --- | --- | --- | --- | --- | --- | --- | --- | --- | --- |
| 2010 | 7.9 | -2.95* | [-3.74, -2.17] | 38.8 | -1.50* | [-1.94, -1.06] | 80.9 | -0.35* | [-0.62, -0.08] |
| 2011 |  |  |  |  |  |  |  |  |  |
| 2012 |  |  |  |  |  |  |  |  |  |
| 2013 | 7.1 | -2.95* | [-3.74, -2.17] | 36.6 | -1.50* | [-1.94, -1.06] | 79.8 | -0.35* | [-0.62, -0.08] |
| 2014 |  |  |  |  |  |  |  |  |  |
| 2015 |  |  |  |  |  |  |  |  |  |
| 2016 | 6.4 | -2.95* | [-3.74, -2.17] | 34.8 | -1.50* | [-1.94, -1.06] | 78.4 | -0.35* | [-0.62, -0.08] |
| 2017 |  |  |  |  |  |  |  |  |  |
| 2018 |  |  |  |  |  |  |  |  |  |
| 2019 | 5.7 | -2.95* | [-3.74, -2.17] | 33.3 | -1.50* | [-1.94, -1.06] | 77.3 | -0.35* | [-0.62, -0.08] |
| 2020 |  |  |  |  |  |  |  |  |  |
| 2021 |  |  |  |  |  |  |  |  |  |
| 2022 |  |  |  |  |  |  |  |  |  |
| 2023 | 5.5 | -2.95* | [-3.74, -2.17] | 32.3 | -1.50* | [-1.94, -1.06] | 77.6 | -0.35* | [-0.62, -0.08] |

**indicates significance at p ≤ 0.05 according to modelled trend in Joinpoint analysis. Blank row in APC indicates Joinpoint in data.
Definitions: Daily drinker: Consumed alcohol daily. Risky drinker (NDSHS): More than 10 standard drinks per week or more than 4 standard drinks on a single day at least once a month on average. Frequent drinker: drinking on 5 or more days per week. Risky drinker (HILDA): Of current drinkers, usually consume 5 or more standard drinks on one occasion.*

Note: The most recent NDSHS survey wave was 2022-23, but for the purposes of table readability this survey wave is reported here as 2023.

Abbreviations: HILDA, Household, Income and Labour Dynamics in Australia survey; NDSHS, National Drug Strategy Household Survey.

| Year | Frequent drinkers, *15+ (HILDA)* | | | | | | Risky drinkers, *15+ (HILDA)* | | | | | |
| --- | --- | --- | --- | --- | --- | --- | --- | --- | --- | --- | --- | --- |
|  | Males | APC | 95% CIs | Females | APC | 95% CIs | Males | APC | 95% CIs | Females | APC | 95% CIs |
| 2010 |  |  |  |  |  |  |  |  |  |  |  |  |
| 2011 | 16.7 | -1.57* | [-2.69, -0.44] | 9.4 | -0.67 | [-1.94, 0.62] | 24.2 | -0.97 | [-1.99, 0.06] | 10.2 | -0.25 | [-1.73, 1.26] |
| 2012 |  |  |  |  |  |  |  |  |  |  |  |  |
| 2013 | 16.3 | -1.57* | [-2.69, -0.44] | 9 | -0.67 | [-1.94, 0.62] | 22.6 | -0.97 | [-1.99, 0.06] | 9.4 | -0.25 | [-1.73, 1.26] |
| 2014 |  |  |  |  |  |  |  |  |  |  |  |  |
| 2015 | 15.5 | -1.57* | [-2.69, -0.44] | 9.1 | -0.67 | [-1.94, 0.62] | 22.7 | -0.97 | [-1.99, 0.06] | 9.9 | -0.25 | [-1.73, 1.26] |
| 2016 |  |  |  |  |  |  |  |  |  |  |  |  |
| 2017 | 15.1 | -1.57* | [-2.69, -0.44] | 8.4 | -0.67 | [-1.94, 0.62] | 23.4 | -0.97 | [-1.99, 0.06] | 9.3 | -0.25 | [-1.73, 1.26] |
| 2018 |  |  |  |  |  |  |  |  |  |  |  |  |
| 2019 | 13.9 | -1.57* | [-2.69, -0.44] | 8.5 | -0.67 | [-1.94, 0.62] | 22.4 | -0.97 | [-1.99, 0.06] | 10.2 | -0.25 | [-1.73, 1.26] |
| 2020 |  |  |  |  |  |  |  |  |  |  |  |  |
| 2021 | 14.8 | -1.57* | [-2.69, -0.44] | 9 | -0.67 | [-1.94, 0.62] | 21.1 | -0.97 | [-1.99, 0.06] | 9.5 | -0.25 | [-1.73, 1.26] |
| 2022 |  |  |  |  |  |  |  |  |  |  |  |  |
| 2023 |  |  |  |  |  |  |  |  |  |  |  |  |

| Year | Current drinkers, *15+ (HILDA)* | | | | | |
| --- | --- | --- | --- | --- | --- | --- |
|  | Males | APC | 95% CIs | Females | APC | 95% CIs |
| 2010 |  |  |  |  |  |  |
| 2011 | 82.5 | -0.43* | [-0.78, -0.08] | 74 | -0.16 | [-0.53, 0.21] |
| 2012 |  |  |  |  |  |  |
| 2013 | 81.1 | -0.43* | [-0.78, -0.08] | 72.7 | -0.16 | [-0.53, 0.21] |
| 2014 |  |  |  |  |  |  |
| 2015 | 79 | -0.43* | [-0.78, -0.08] | 71.8 | -0.16 | [-0.53, 0.21] |
| 2016 |  |  |  |  |  |  |
| 2017 | 79.2 | -0.43* | [-0.78, -0.08] | 71.8 | -0.16 | [-0.53, 0.21] |
| 2018 |  |  |  |  |  |  |
| 2019 | 79.4 | -0.43* | [-0.78, -0.08] | 72 | -0.16 | [-0.53, 0.21] |
| 2020 |  |  |  |  |  |  |
| 2021 | 78.6 | -0.43* | [-0.78, -0.08] | 72.8 | -0.16 | [-0.53, 0.21] |
| 2022 |  |  |  |  |  |  |
| 2023 |  |  |  |  |  |  |

#### Part 2. Alcohol-Related Harm Measures

Figure indicating modelled Joinpoint trends reporting additional measures of deaths related to alcohol

Figure indicating modelled Joinpoint trends reporting additional measures of hospitalisations and related harms due to alcohol

*Definitions: Alcohol-attributable deaths: Deaths where the underlying cause is either entirely or fractionally caused by alcohol according to calculated Australian-specific alcohol-attributable fractions (including wholly or partially). Alcohol-related injury deaths: deaths with either a principal diagnosis of injury and additional in-scope diagnosis relating to alcohol, or external cause of injury relating to alcohol.Alcohol-induced deaths: deaths where the underlying cause was directly attributable to alcohol use.*
Note: Observed values are indicated by scatter points, and modelled trends indicated by line/dashed line. Statistically significant trends are indicated by solid lines and trends that are not statistically significant are indicated by dashed lines. A black cross indicates a joinpoint in the data.

*Definitions: Alcohol-attributable hospitalisations: Hospitalisations based on either primary diagnoses or external causes relating to alcohol use according to calculated Australian-specific alcohol-attributable fractions (including wholly or partially). Alcohol-related injury hospitalisations: Hospitalisations with either a principal diagnosis of injury and additional in-scope diagnosis relating to alcohol, or external cause of injury relating to alcohol.*
Note: Observed values are indicated by scatter points, and modelled trends indicated by line/dashed line. Statistically significant trends are indicated by solid lines and trends that are not statistically significant are indicated by dashed lines. A black cross indicates a joinpoint in the data.

**Table C.** Observed values, annual percentage change (APC), and confidence intervals (CI) indicating trends in deaths and hospitalisations related to alcohol (age-standardised rates per 100,000 population), sourced from the NAIP, NIDIP and AIHW (continued on next page)

| Year | Alcohol-attributable deaths, *15+ population (NAIP)* | | | | | | Alcohol-induced deaths,  *entire population (NIDIP)* | | | Alcohol-related injury deaths,  *entire population (AIHW)* | | |
| --- | --- | --- | --- | --- | --- | --- | --- | --- | --- | --- | --- | --- |
|  | Wholly | APC | 95% CIs | Partially | APC | 95% CIs | Total | APC | 95% CIs | Total | APC | 95% CIs |
| 2010 |  |  |  |  |  |  | 5.00 | 1.60* | [0.72, 2.50] |  |  |  |
| 2011 | 6.14 | 0.80 | [-0.79, 2.41] | 11.15 | 5.18* | [4.54, 5.82] | 4.71 | 1.60* | [0.72, 2.50] | 4.8 | 16.75* | [5.93, 28.69] |
| 2012 | 5.9 | 0.80 | [-0.79, 2.41] | 11.68 | 5.18* | [4.54, 5.82] | 4.52 | 1.60* | [0.72, 2.50] | 5 | 16.75* | [5.93, 28.69] |
| 2013 | 6.38 | 0.80 | [-0.79, 2.41] | 12.71 | 5.18* | [4.54, 5.82] | 4.85 | 1.60* | [0.72, 2.50] | 6.8 | 16.75* | [5.93, 28.69] |
| 2014 | 6.68 | 0.80 | [-0.79, 2.41] | 12.73 | 5.18* | [4.54, 5.82] | 5.04 | 1.60* | [0.72, 2.50] | 7.5 | 16.75* | [5.93, 28.69] |
| 2015 | 6.86 | 0.80 | [-0.79, 2.41] | 13.52 | 5.18* | [4.54, 5.82] | 5.24 | 1.60* | [0.72, 2.50] | 8.6 |  |  |
| 2016 | 6.4 | 0.80 | [-0.79, 2.41] | 14.41 | 5.18* | [4.54, 5.82] | 4.83 | 1.60* | [0.72, 2.50] | 8.3 | 4.03 | [-2.89, 11.45] |
| 2017 | 6.93 | 0.80 | [-0.79, 2.41] | 15.01 | 5.18* | [4.54, 5.82] | 5.28 | 1.60* | [0.72, 2.50] | 9.6 | 4.03 | [-2.89, 11.45] |
| 2018 | 6.21 | 0.80 | [-0.79, 2.41] | 15.71 | 5.18* | [4.54, 5.82] | 4.69 | 1.60* | [0.72, 2.50] | 10.5 | 4.03 | [-2.89, 11.45] |
| 2019 | 6.39 | 0.80 | [-0.79, 2.41] | 17.13 |  |  | 4.96 | 1.60* | [0.72, 2.50] | 10.8 | 4.03 | [-2.89, 11.45] |
| 2020 | 6.77 |  |  | 16.41 | 0.32 | [-2.44, 3.15] | 5.36 | 1.60* | [0.72, 2.50] | 9.7 | 4.03 | [-2.89, 11.45] |
| 2021 | 7.37 | 10.91 | [-6.80, 31.99] | 17.1 | 0.32 | [-2.44, 3.15] | 5.63 | 1.60* | [0.72, 2.50] |  |  |  |
| 2022 | 8.26 | 10.91 | [-6.80, 31.99] | 16.79 | 0.32 | [-2.44, 3.15] | 6.21 | 1.60* | [0.72, 2.50] |  |  |  |
| 2023 |  |  |  |  |  |  | 5.76 | 1.60* | [0.72, 2.50] |  |  |  |

**indicates significance at p ≤ 0.05 according to modelled trend in Joinpoint analysis. Blank row in APC indicates Joinpoint in data.*

*Definitions: Alcohol-attributable deaths: Deaths where the underlying cause is either entirely or fractionally caused by alcohol according to calculated Australian-specific alcohol-attributable fractions (including wholly or partially). Alcohol-induced deaths: Deaths where the underlying cause was directly attributable to alcohol use. Alcohol-related deaths: Including both deaths directly attributable to alcohol and deaths where alcohol was listed as an associated cause. Alcohol-attributable hospitalisations: Hospitalisations based on either primary diagnoses or external causes relating to alcohol use according to calculated Australian-specific alcohol-attributable fractions (including wholly or partially). Alcohol-related injury hospitalisations: Hospitalisations with either a principal diagnosis of injury and additional in-scope diagnosis relating to alcohol, or external cause of injury relating to alcohol.*
Note: The AIHW reports according to financial years (e.g., 2011-12). For table readability, these are reported here according to the later part of the year (e.g., 2012).

| Year | Alcohol-attributable hospitalisations, *15+ population (NAIP)* | | | | | | Alcohol-related injury hospitalisations^a^,  *entire population (AIHW)* | | |
| --- | --- | --- | --- | --- | --- | --- | --- | --- | --- |
|  | Wholly | APC | 95% CIs | Partially | APC | 95% CIs | Total | APC | 95% CIs |
| 2010 |  |  |  |  |  |  |  |  |  |
| 2011 | 315.99 | 1.37* | [0.71, 2.04] | 152.9 | 2.40* | [1.45, 3.36] | 133.5 | -1.86 | [-4.90, 1.27] |
| 2012 | 319.5 | 1.37* | [0.71, 2.04] | 159.47 | 2.40* | [1.45, 3.36] | 133.8 | -1.86 | [-4.90, 1.27] |
| 2013 | 314.05 | 1.37* | [0.71, 2.04] | 148.88 | 2.40* | [1.45, 3.36] | 129.9 | -1.86 | [-4.90, 1.27] |
| 2014 | 317.27 | 1.37* | [0.71, 2.04] | 150.03 | 2.40* | [1.45, 3.36] | 124.4 | -1.86 | [-4.90, 1.27] |
| 2015 | 320.75 | 1.37* | [0.71, 2.04] | 163.07 | 2.40* | [1.45, 3.36] | 125.4 |  |  |
| 2016 | 327.8 | 1.37* | [0.71, 2.04] | 175.63 | 2.40* | [1.45, 3.36] | 132.0 | 4.77 | [-5.14. 15.73] |
| 2017 | 330.7 | 1.37* | [0.71, 2.04] | 185.68 | 2.40* | [1.45, 3.36] | 136.2 | 4.77 | [-5.14. 15.73] |
| 2018 | 337.03 | 1.37* | [0.71, 2.04] | 189.36 | 2.40* | [1.45, 3.36] | 122.9 | *N/A* |  |
| 2019 | 345.56 | 1.37* | [0.71, 2.04] | 187.71 | 2.40* | [1.45, 3.36] | 122.1 | *N/A* |  |
| 2020 | 333.31 | 1.37* | [0.71, 2.04] | 186.29 | 2.40* | [1.45, 3.36] | 115.8 | *N/A* |  |
| 2021 | 386.83 | 1.37* | [0.71, 2.04] | 189.45 | 2.40* | [1.45, 3.36] |  |  |  |
| 2022 | 351.57 | 1.37* | [0.71, 2.04] | 183.93 | 2.40* | [1.45, 3.36] |  |  |  |
| 2023 |  |  |  |  |  |  |  |  |  |

^a^The AIHW recommends against comparing 2010-2017 and 2018-2020 trends due to changes in data collection methods, as indicated by the line in the table. Trends from 2018-2020 could not be analysed via Joinpoint due to lack of data points, and are therefore only reported here.

Abbreviations: AIHW, Australian Institute of Health and Welfare; NAIP, National Alcohol Indicators Project; NIDIP, National Illicit Drug Indicators Project.

#### Part 3. Summary Results

**Table D.** Annual percentage change for supplementary measures of alcohol consumption and harm per year, according to Joinpoint analyses.

| ***Consumption measures*** | | | | | | | | | | | | | | |
| --- | --- | --- | --- | --- | --- | --- | --- | --- | --- | --- | --- | --- | --- | --- |
| **Measure** | **2010** | **2011** | **2012** | **2013** | **2014** | **2015** | **2016** | **2017** | **2018** | **2019** | **2020** | **2021** | **2022** | **2023** |
| Daily drinkers  *15+ (%), NDSHS^c^* | **-2.95** | **-2.95** | **-2.95** | **-2.95** | **-2.95** | **-2.95** | **-2.95** | **-2.95** | **-2.95** | **-2.95** | **-2.95** | **-2.95** | **-2.95** | **-2.95** |
| Risky drinkers  *15+ (%), NDSHS^c^* | **-1.50** | **-1.50** | **-1.50** | **-1.50** | **-1.50** | **-1.50** | **-1.50** | **-1.50** | **-1.50** | **-1.50** | **-1.50** | **-1.50** | **-1.50** | **-1.50** |
| Frequent drinkers *Males 15+ (%), HILDA^c^* |  | **-1.57** | **-1.57** | **-1.57** | **-1.57** | **-1.57** | **-1.57** | **-1.57** | **-1.57** | **-1.57** | **-1.57** | **-1.57** |  |  |
| Frequent drinker  *Females 15+ (%), HILDA^c^* |  | -0.67 | -0.67 | -0.67 | -0.67 | -0.67 | -0.67 | -0.67 | -0.67 | -0.67 | -0.67 | -0.67 |  |  |
| Risky drinker  *Males 15+ (%), HILDA^c^* |  | -0.97 | -0.97 | -0.97 | -0.97 | -0.97 | -0.97 | -0.97 | -0.97 | -0.97 | -0.97 | -0.97 |  |  |
| Risky drinker  *Females 15+ (%), HILDA^c^* |  | -0.25 | -0.25 | -0.25 | -0.25 | -0.25 | -0.25 | -0.25 | -0.25 | -0.25 | -0.25 | -0.25 |  |  |
| Current drinkers  *Males 15+ (%), HILDA^c^* |  | **-0.43** | **-0.43** | **-0.43** | **-0.43** | **-0.43** | **-0.43** | **-0.43** | **-0.43** | **-0.43** | **-0.43** | **-0.43** |  |  |
| Current drinkers  *Females 15+ (%), HILDA^c^* |  | -0.16 | -0.16 | -0.16 | -0.16 | -0.16 | -0.16 | -0.16 | -0.16 | -0.16 | -0.16 | -0.16 |  |  |
| Current drinkers  *15+ (%), NDSHS^c^* | **-0.35** | **-0.35** | **-0.35** | **-0.35** | **-0.35** | **-0.35** | **-0.35** | **-0.35** | **-0.35** | **-0.35** | **-0.35** | **-0.35** | **-0.35** | **-0.35** |
| ***Alcohol-related harm measures*** | | | | | | | | | | | | | | |
| **Measure** | **2010** | **2011** | **2012** | **2013** | **2014** | **2015** | **2016** | **2017** | **2018** | **2019** | **2020** | **2021** | **2022** | **2023** |
| Alcohol-attributable deaths  *15+ (per 100,000) - Wholly, NAIP^a^* |  | 0.80 | 0.80 | 0.80 | 0.80 | 0.80 | 0.80 | 0.80 | 0.80 | 0.80 | 10.91 | 10.91 | 10.91 |  |
| Alcohol-attributable deaths  *15+ (per 100,000) - Partially, NAIP^a^* |  | **5.18** | **5.18** | **5.18** | **5.18** | **5.18** | **5.18** | **5.18** | **5.18** | 0.32 | 0.32 | 0.32 | 0.32 |  |
| Alcohol-induced deaths  *entire pop. (per 100,000)*, *NIDIP^a^* | 1.60 | 1.60 | 1.60 | 1.60 | 1.60 | 1.60 | 1.60 | 1.60 | 1.60 | 1.60 | 1.60 | 1.60 | 1.60 | 1.60 |
| Alcohol-related injury deaths  *entire pop. (per 100,000), AIHW^b^* |  | **16.75** | **16.75** | **16.75** | **16.75** | **16.75** | 4.03 | 4.03 | 4.03 | 4.03 | 4.03 |  |  |  |
| Alcohol-attributable hospitalisations  *15+ (per 100,000) - Wholly, NAIP^a^* |  | **1.37** | **1.37** | **1.37** | **1.37** | **1.37** | **1.37** | **1.37** | **1.37** | **1.37** | **1.37** | **1.37** | **1.37** |  |
| Alcohol-attributable hospitalisations  *15+ (per 100,000) - Partially, NAIP^a^* |  | **2.40** | **2.40** | **2.40** | **2.40** | **2.40** | **2.40** | **2.40** | **2.40** | **2.40** | **2.40** | **2.40** | **2.40** |  |
| Alcohol-related injury hospitalisations  *entire pop. (per 100,000), AIHW^b^* |  | -1.86 | -1.86 | -1.86 | -1.86 | 4.78 | 4.78 | 4.78 |  |  |  |  |  |  |

*Note: Bolded black text indicates significant trend at p ≤ 0.05, non-bolded text indicates non-significant trend. Double-lined cell border indicates Joinpoint. Blue cells indicate decreasing trends, red cells indicated increasing trends. Strength of shading indicates greater trend (i.e., annual percentage change* *of 0-5 are lightest, 5-10 are medium, and 15+ darkest shades). Some data sources (such as NDSHS) are not annual data (see Table A for further information), however, Joinpoint provides annual values indicating percentage change.*

*^a^indicates measure with 12-16 data points and a maximum of 2 joinpoints tested*

*^b^indicates measure with 7-11 data points and a maximum of 1 joinpoint tested*

*^c^indicates measure with 0-6 data points and 0 joinpoints tested*

Abbreviations: AIHW, Australian Institute of Health and Welfare; HILDA, Household, Income and Labour Dynamics in Australia survey; NAIP, National Alcohol Indicators Project; NDSHS, National Drug Strategy Household Survey; NIDIP, National Illicit Drug Indicators Project.

**Table E.** Table indicating average annual percentage change (APC) for supplementary measures with a time period spanning 2012-2021.

| **Measure type** | **Measurement** | **Average APC** | **Confidence intervals** |
| --- | --- | --- | --- |
| **Survey alcohol consumption** | Daily drinker (NDSHS) | -2.95* | [-3.74, -2.17] |
|  | Risky drinker (NDSHS) | -1.50* | [-1.94, -1.06] |
|  | Current drinker (NDSHS) | -0.35* | [-0.62, 0.08] |
|  | Frequent drinker (HILDA) |  |  |
|  | *Males* | -1.57* | [-2.69, -0.44] |
|  | *Females* | -0.67 | [-1.94, 0.62] |
|  | Risky drinker (HILDA) |  |  |
|  | *Males* | -0.97 | [-1.99, 0.06] |
|  | *Females* | -0.25 | [-1.73, 1.26] |
|  | Current drinker (HILDA) |  |  |
|  | *Males* | -0.43* | [-0.78, -0.08] |
|  | *Females* | -0.16 | [-0.53, 0.21] |
| **Alcohol-related harms** | Alcohol-induced deaths (NIDIP) | 1.60* | [0.72, 2.50] |

*Note: * indicates significant average APC at p ≤ 0.05. Alcohol-related injury hospitalisations and alcohol-related injury deaths are not included in this table as they did not have data for the entire 2012-2021 period for comparison.*

Abbreviations: HILDA, Household, Income and Labour Dynamics in Australia survey; NDSHS, National Drug Strategy Household Survey; NIDIP, National Illicit Drug Indicators Project.

## Table S1. Information on data sources and limitations

| **Non-Survey Alcohol Consumption Measures** | | | | |
| --- | --- | --- | --- | --- |
| **Name [range]** | **Description** | **Unit of measurement** | **Coding or operational definition** | **Limitations of source** |
| **Per Capita Consumption**  **[2010/11-2022/23]** | Total alcohol consumption per capita (in litres) based on each major drink types [1] | Litres per person per day | A range of sources are used to calculate total consumption, including excise data, import clearance records, and financial information. | - Data is limited due to reliance on tax data and lack of availability of sales data to inform estimates - Wine and cider estimates rely on assumptions because their alcohol content cannot be estimated from tax. - A new method for estimating wine consumption was used from 2014/15 onwards, however, has been validated to ensure time series comparability. |
| **Per Capita Consumption via Wastewater Estimates**  **[2016-2024]** | Estimates of total alcohol consumption per capita. Samples are collected every 2-4 months from regional and capital city sites across the country covering 43-61% of the Australian population [2]. | Litres per person per day | Estimates were extracted by visually inspecting graphs within the 23 reports published from 2016-2023, with averages for each year calculated (litres per 1000 per day), and transformed to a measure of litres per person per year. | - While this objective source avoids response-related biases of surveys, there are some uncertainties regarding sampling and estimation from detection of biomarkers in wastewater [3], making it best used as a complementary measure alongside other sources. |
| **Survey Alcohol Consumption Measures** | | | | |
| **Name [range]** | **Description** | **Unit of measurement** | **Coding or operational definition** | **Limitations of source** |
| **National Drug Strategy Household Survey**  **[2010-2023]** | A nationally representative household survey employing stratified, random sampling capturing the population aged 15 and older [4] | a) Daily drinker b) Risky drinker c) High (weekly) risky drinker d) High (monthly) risky drinker e) Current drinker | a) Percentage of population consuming any alcohol daily b) Percentage of population consuming more than 10 standard drinks per week or more than 4 standard drinks on a single day at least once a month on average in the past 12 months c) Percentage of population consuming more than 10 standard drinks per week on average in the past 12 months d) Percentage of population consuming more than 4 standard drinks on a single day at least once a month over the previous 12 months e) Percentage of population who consumed alcohol in the past 12 months | - Average survey response rate was 48.8% (ranging from 50.6% in 2010 to lower in more recent years, with a response rate of 43.9% in 2022-23). - Response rates are calculated based circumstances where contact has been established (i.e., households contacted with no response are not included as ‘non-responders’). For 2022-23, if the entire sample is used to calculate responses, the response rate would be reduced to 30.6%. - Populations excluded from sampling include non-private dwellings (e.g., hotels), institutional settings (e.g., hospitals, prisons, university halls, etc), and homeless populations. - The sample has been designed to provide a random sample of households within each geographic stratum, with weights applied to account for selection probabilities, adjusting for non-response, under-sampled or over-sampled strata, and other known differences between the sample and entire population. Population estimates used for weighting were based on the latest available age profile using the published Australian Bureau of Statistics Estimated Resident Population data, with the stratum level population estimates projected from the regional Estimated Resident Population series. - Data are self-reported, thus respondents may not accurately report information. |
| **Household, Income and Labour Dynamics in Australia (HILDA) Survey**  **[2011-2021]** | A household-based panel study employing a multi-stage sampling approach to ensure representativeness across private dwellings in Australia, capturing the population aged 15 and older. To account for drop-out (due to the panel-based nature of the survey), a top-up sample of households was added to the survey in 2011 [5] | a) Frequent drinker b) Risky drinker c) Frequent risky drinker d) Current drinker | a) Percentage of population drinking on 5 or more days per week (by sex) in the previous 12 months b) Percentage of population who were current drinkers consuming 5 or more standard drinks on one occasion (by sex) in the previous 12 months c) Percentage of population who were current drinkers consuming at least 5 (if female) or 7 (if male) standard drinks on one occasion at least 2 or 3 times per month (by sex) in the previous 12 months d) Percentage of population who reported ever drinking alcohol (by sex) in the previous 12 months | - Response rates range depending on survey year and sample composition, from approximately 60% in 2021 for new entrants in the top-up sample (response rates were noted to drop during pandemic years) to approximately 96% in 2019 for previous wave respondents in the original sample. - Populations excluded from sampling include those living in remote and sparsely populated areas, those living in non-private dwellings (e.g., hotels), and residents of institutions (e.g., hospitals, health care institutions, military/police installations, correctional institutions, convents and monasteries) - Non-response and attrition are particularly an issue for panel surveys – leading to such panels becoming increasingly less representative of the wider population. However, weights are created to adjust for this. The HILDA Survey uses person-level weights to adjust for unequal probabilities of selection and non-response. In all waves, survey weights sum to the total Australian population, inclusive of new immigrants. For longitudinal analyses, longitudinal responding person weights are applied; these adjust for attrition from the original sample and are benchmarked to Australian population totals based on Australian Bureau of Statistics population estimates. - Data are self-reported, thus respondents may not accurately report information. |
| **National Health Survey**  **[2012-2022]** | A nationally representative survey employing random sampling of private residences in Australia, capturing the population aged 18 and older [6] | a) Single-occasion risky drinker b) Long-term risky drinker | a) Percentage of population who exceeded guideline “no more than 4 standard drinks on a single occasion” in the previous 12 months b) Percentage of population who exceeded guideline “no more than 2 standard drinks per day” in the previous 12 months | - The 2020-21 survey wave faced significant data collection changes. - Response rates have ranged from 56.7% in 2022 (which was notably low due to the effects of the pandemic) to 84.8% in 2014/15 [7]. - The following were excluded from sampling: visitors, non-private dwelling occupants (e.g., hotels, hospitals, caravan parks), those in very remote areas, discrete Aboriginal & Torres Strait Islander communities, and households where residents are less than 18 years old. - The NHS assigns each respondent a weight based on their probability of selection. Weights are then calibrated to align with independent estimates of the in-scope population based on the estimated resident population, referred to as ‘benchmarks.’ These benchmarks use additional information about the population to ensure that the sample is representative and that survey estimates reflect the distribution of the whole population, not the sample. - Data are self-reported, thus respondents may not accurately report information. |
| **Alcohol-Related Harms** | | | | |
| **Name [range]** | **Description** | **Unit of measurement** | **Coding or operational definition** | **Limitations of source** |
| **Alcohol-Related Deaths**  **[2010-2022]** | Deaths directly attributable to alcohol and where alcohol is listed as an associated cause, sourced from the National Mortality Database for the entire population [8] | Age standardised rate (per 100,000) | Alcohol-related deaths are defined as those that are alcohol-induced (determined according to their intent) as well as where alcohol is an associated cause of death.  The following ICD-10 codes were used to identify alcohol-induced deaths: T51.0, T51.1, T51.2, T51.3, T51.8, T51.9  Codes used for broader alcohol-related deaths could not be identified. | - Reporting practices may change over time, as the ICD is revised periodically to incorporate changes in the medical field. - Deaths data from 2021 (revised) and 2022 (preliminary revised) are subject to further revision. |
| **Alcohol-Induced Deaths**  **[2010-2023]** | Deaths with an underlying cause directly attributable to alcohol for the entire population [9]  Mortality estimates are calculated from the Cause of Death Unit Record File, which contains all deaths occurring and registered in Australia managed by the Australian Coordinating Registry on behalf of the Registries of Births, Deaths and Marriages, Coroners, and the National Coronial Information System | Age standardised rate (per 100,000) | The following ICD-10-AM codes were used to identify alcohol-induced death cases:  E24.4; F10; G31.2; G62.1; G72.1; I42.6; K29.2; K70; K85.2; K86.0; R78.0; X45; X65; Y15 | - 2023 data are preliminary revised, and data for 2022 are revised, meaning both will be subject to further revision. - Data excludes accidents, homicides, and other causes indirectly related to alcohol use. - Data excludes newborn deaths associated with maternal alcohol use. |
| **Alcohol-Attributable Deaths**  **[2011-2022]** | Deaths where the underlying cause is wholly (e.g., alcohol toxicity) or partially (e.g., breast cancer) attributable to alcohol, with the latter estimated using Australian age- and sex-specific alcohol-attributable fractions. Estimates calculated for the population aged 15 and over [10]  Death data were provided by the Australian Coordinating Registry at the Queensland Registry of Births, Deaths and Marriages. | Age standardised rate (per 100,000) | Alcohol-attributable fractions were calculated in an open access online alcohol harm estimator tool (International Model of Alcohol Harms and Policies, InterMAHP v2.1) [11]  Alcohol-attributable conditions are defined as those utilising the following ICD-10-AM codes:  Cancers: C01-C06.9; C09-C10.9; C12-C14.9; C15; C16; C18-20; C22; C25; C32; C50  Cardiovascular Diseases: I10-I15.9; I20-I25.9; I42.6; I47.1; I47.9; I48; I60-I62.9; I69.0-I69.2; G45; I63; I65-I67.9; I69.3; I64, I69.4, I69.8; I85; I98.20; I98.21  Digestive Diseases: K29.2; K74.3-K74.6; K76.0; K76.9; K70; K85; K86.1; K86.0  Endocrine Conditions: E11; E24.4  Infectious Diseases: A15.0-A19.9; B20.0-B24.9; Z21; J09.0-J22.9  Injuries (Unintentional): V12.3-V12.9; V13.3-V13.9; V14.3-V14.9; V19.4-V19.6; V19.9; V20.3-V20.9; V21.3-V21.9; V22.3-V22.9; V23.3-V23.9; V24.3-V24.9; V25.3-V25.9; V26.3-V26.9; V27.3-V27.9; V28.3-V28.9; V29.4-V29.9; V30.4-V30.9; V31.4-V31.9; V32.4-V32.9; V33.4-V33.9; V34.4-V34.9; V35.4-V35.9; V36.4-V36.9; V37.4-V37.9; V38.4-V38.9; V39.4-V39.9; V40.4-V40.9; V41.4-V41.9; V42.4-V42.9; V43.4-V43.9; V44.4-V44.9; V45.4-V45.9; V46.4-V46.9; V47.4-V47.9; V48.4-V48.9; V49.4-V49.9; V50.4-V50.9; V51.4-V51.9; V52.4-V52.9; V53.4-V53.9; V54.4-V54.9; V55.4-V55.9; V56.4-V56.9; V57.4-V57.9; V58.4-V58.9; V59.4-V59.9; V60.4-V60.9; V61.4-V61.9; V62.4-V62.9; V63.4-V63.9; V64.4-V64.9; V65.4-V65.9; V66.4-V66.9; V67.4-V67.9; V68.4-V68.9; V69.4-V69.9; V70.4-V70.9; V71.4-V71.9; V72.4-V72.9; V73.4-V73.9; V74.4-V74.9; V75.4-V75.9; V76.4-V76.9; V77.4-V77.9; V78.4-V78.9; V79.4-V79.9; V80.3-V80.5; V81.1; V82.1; V82.9; V83.0-V83.3;V84.0-V84.3;V85.0-V85.3;V86.0-V86.3; V87.0-V87.9; V89.2; V89.3; V89.9; V02.1-V02.9; V03.1-V03.9; V04.1-V04.9; V09.2-V09.3; V06.1; W00-W19.9; W65-W74.9; X00-X09.9; X45; Y15; W78; W79; W24-W31.9; W45; W49; W60  Injuries (Intentional): X60.0-X64.9; X66-X84.9; Y87; X65; X85-X89.9; Y00-Y09.9; Y87.1  Neuropsychiatric Conditions: F10.0-F10.9; G31.2; G40; G41; G62.1; G72.1 | - Deaths data (2011-2022) were preliminary at time of data request, thus results may underreport alcohol-attributable deaths where cause of death was not confirmed until a coronial inquiry was completed - Records where sex was ‘other’ or missing were excluded; records where age was missing was also excluded |
| **Alcohol-Related Injury Deaths**  **[2011-2020]** | Injury-specific deaths with either a principal diagnosis of injury and additional in-scope diagnosis relating to alcohol, or external cause of injury relating to alcohol for the entire population [12] | Age standardised rate (per 100,000) | Inclusion criteria for an alcohol-related injury death:   - Criterion 1 (An alcohol-related underlying cause of death of X45 or X65 or Y15)   OR   - Criterion 2 (An injury-related cause of death and an injury-related external cause of death and an in-scope alcohol-related cause of death)   OR   - Criterion 3 (An in-scope alcohol-related injury cause of death and an injury-related external cause of death)   OR   - Criterion 4 (An injury-related cause of death and an in-scope alcohol-related external cause of death)   Injury is defined by cause-of-death codes in the ICD-10 classification system within the range S00–T75 and T79.  An alcohol-related condition included one of the following codes: F10.0, F10.1, F10.2, R78.0, T51.0, T51.9, X45, X65, Y15, Y90, Z72.1 | - Potential issues with data quality in that the data may include cases where alcohol was consumed after the injury event, or cases where alcohol was consumed but did not contribute to the injury event. Additionally, may exclude additional cases where alcohol was not detected, divulged, or considered relevant and therefore not recorded. - Does not include emergency department datasets due to unreliability of national emergency department data - Due to time taken to finalise coronial investigations, there may be a delay in complete reports of deaths |
| **Alcohol-Related Hospitalisations**  **[2011-2023]** | Hospitalisations where an alcohol-related diagnosis code was identified in the principal diagnosis field, capturing the entire population [13]  Estimates are calculated from the AIHW’s National Hospital Morbidity Database of episode-level records from Australian public and private hospitals coded based on the ICD-10-AM diagnoses. | Age standardised rate (per 100,000) | The following ICD-10-AM codes were used to identify alcohol-related hospitalisation cases:  T51.0 – T51.9; F10.0-F10.9; E24.4; E52; G31.2; G62.1; G72.1; I42.6; K29.2; K29.20; K29.21; K70 (code starting with the characters); K85.2; K86.0; Z71.4; R78.0 | - Estimates of hospitalisations in Tasmania for 2015-16 may be underestimated due to limited data - Hospitalisation data for 2011/12-2012/13 in Victoria may be underestimated due to differences in reporting for emergency department stays |
| **Alcohol-Attributable Hospitalisations**  **[2011-2022]** | Hospitalisations where primary diagnoses or external causes are related to alcohol use using alcohol-attributable fractions, capturing the population aged 15 and over [10]  Estimates are calculated from the AIHW’s National Hospital Morbidity Database of episode-level records from Australian public and private hospitals coded based on ICD-10-AM diagnoses. | Age standardised rate (per 100,000) | Alcohol-attributable fractions were calculated in an open access online alcohol harm estimator tool (International Model of Alcohol Harms and Policies, InterMAHP v2.1) [11]  Alcohol-attributable conditions are defined as those utilising the following ICD-10-AM codes:  Cancers: C01-C06.9; C09-C10.9; C12-C14.9; C15; C16; C18-20; C22; C25; C32; C50  Cardiovascular Diseases: I10-I15.9; I20-I25.9; I42.6; I47.1; I47.9; I48; I60-I62.9; I69.0-I69.2; G45; I63; I65-I67.9; I69.3; I64, I69.4, I69.8; I85; I98.20; I98.21  Digestive Diseases: K29.2; K74.3-K74.6; K76.0; K76.9; K70; K85; K86.1; K86.0  Endocrine Conditions: E11; E24.4  Infectious Diseases: A15.0-A19.9; B20.0-B24.9; Z21; J09.0-J22.9  Injuries (Unintentional): V12.3-V12.9; V13.3-V13.9; V14.3-V14.9; V19.4-V19.6; V19.9; V20.3-V20.9; V21.3-V21.9; V22.3-V22.9; V23.3-V23.9; V24.3-V24.9; V25.3-V25.9; V26.3-V26.9; V27.3-V27.9; V28.3-V28.9; V29.4-V29.9; V30.4-V30.9; V31.4-V31.9; V32.4-V32.9; V33.4-V33.9; V34.4-V34.9; V35.4-V35.9; V36.4-V36.9; V37.4-V37.9; V38.4-V38.9; V39.4-V39.9; V40.4-V40.9; V41.4-V41.9; V42.4-V42.9; V43.4-V43.9; V44.4-V44.9; V45.4-V45.9; V46.4-V46.9; V47.4-V47.9; V48.4-V48.9; V49.4-V49.9; V50.4-V50.9; V51.4-V51.9; V52.4-V52.9;V53.4-V53.9; V54.4-V54.9;V55.4-V55.9;V56.4-V56.9;V57.4-V57.9;V58.4-V58.9; V59.4-V59.9;V60.4-V60.9;V61.4-V61.9;V62.4-V62.9;V63.4-V63.9; V64.4-V64.9;V65.4-V65.9;V66.4-V66.9; V67.4-V67.9;V68.4-V68.9; V69.4-V69.9;V70.4-V70.9;V71.4-V71.9;V72.4-V72.9;V73.4-V73.9; V74.4-V74.9;V75.4-V75.9;V76.4-V76.9; V77.4-V77.9;V78.4-V78.9; V79.4-V79.9;V80.3-V80.5; V81.1; V82.1; V82.9; V83.0-V83.3;V84.0-V84.3;V85.0-V85.3;V86.0-V86.3; V87.0-V87.9; V89.2; V89.3; V89.9; V02.1-V02.9; V03.1-V03.9; V04.1-V04.9; V09.2-V09.3; V06.1; W00-W19.9; W65-W74.9; X00-X09.9; X45; Y15; W78; W79; W24-W31.9; W45; W49; W60  Injuries (Intentional): X60.0-X64.9; X66-X84.9; Y87; X65; X85-X89.9; Y00-Y09.9; Y87.1  Neuropsychiatric Conditions: F10.0-F10.9; G31.2; G40; G41; G62.1; G72.1 | - Health conditions related to the impacts of maternal consumption on the foetus, such as foetal alcohol spectrum disorder, have been excluded - Records where sex was ‘other’ or missing were excluded; records were age was missing was also excluded |
| **Alcohol-Related Injury Hospitalisations**  **[2011-2017]** | Injury-specific hospitalisations with either a principal diagnosis of injury and additional in-scope diagnosis relating to alcohol, or principal diagnosis of acute alcohol intoxication and additional diagnosis of injury, capturing data for the entire population [12]  Estimates are calculated from the AIHW’s National Hospital Morbidity Database (NHMD) of episode-level records from Australian public and private hospitals coded based on ICD-10-AM diagnoses. | Rate (per 100,000) | Inclusion criteria for an alcohol-related injury hospitalisation are based on the following:   - Criterion 1: A principal diagnosis of injury (S00-T75; T79) and an additional in-scope diagnosis relating to alcohol use (F10.0, F10.1, F10.2, R78.0, T51.0, T51.9, Z72.1) or an external cause of injury relating to alcohol use (X45, X65, Y15, Y90)   OR   - Criterion 2: A principal diagnosis of acute alcohol intoxication (F10.0) and an additional diagnosis of injury (S00-T75; T79) | - Potential issues with data quality in that the data may include cases where alcohol was consumed after the injury event, or cases where alcohol was consumed but did not contribute to the injury event. Additionally, may exclude additional cases where alcohol was not detected, divulged, or considered relevant and therefore not recorded. - Does not include emergency department datasets due to unreliability of national emergency department data - Data for this measure exist from 2011-2020, however, the AIHW recommend against comparing the 2018-2020 data to earlier trends due to a break in the time series as a result of changes in data collection methods. |
| **Alcohol Ambulance Attendances**  **[2015-2022]** | Ambulance attendances with alcohol involvement capturing the population attended by ambulances [14] | Rate (per 100,000) | Data is extracted from electronic data collection systems used by paramedics. Trained clinical coders manually examine each record to identify the substance involved.  Alcohol involvement is identified by paramedic clinical assessment, patient self-report, information from third parties, and/or evidence from the scene | - Western Australia and South Australia are not included - Data availability for the remaining jurisdictions varies throughout the time period |
| **Closed Treatment Episodes for Alcohol**  **[2013/14-2022/23]** | Treatment episodes that were treatment for one’s own use of alcohol, capturing the population with treatment episodes [15]  Data is based on the Alcohol and Other Drug Treatment Services National Minimum Dataset (AODTS NMDS), comprising data from publicly funded government and non-government agencies providing specialist alcohol and other drug treatment services | Percentage of alcohol treatment episodes (compared to treatment for other substances) | Treatment episodes for alcohol are identified where the treatment is for one’s own use of alcohol where alcohol was listed as the principal drug of concern. This is determined due to being the main treatment type, reason for cessation, or source of referral. Treatment episodes are defined where the treatment end date fell within the reporting period.  Crude rates (per 100,000) for treatment episodes for alcohol were calculating by the authors based on the number of treatment episodes for alcohol & population estimates for that year sourced from the Australian Bureau of Statistics [16]. | - Excluded treatment settings include: not-for-profit or private treatment agencies that do not receive public funding; treatment units in acute care or psychiatric hospitals providing treatment only to admitted patients; prisons, correctional facilities and detention centres; primary health-care services; health promotion services; or accommodation services - For 2014-15 and 2015-16 reporting periods, sector reforms and system issues in some jurisdictions affected the number of in-scope agencies that reported, leading to an under-count of the number of closed treatment episodes reported for these years. Results across reporting years should be interpreted with caution. - Treatment episodes reflect at least partly the availability and uptake of treatment meaning increases do not necessarily represent increasing incidence of alcohol use disorder. |
| **Positive Alcohol Random Breath Tests**  **[2010-2022]** | Proportion of random breath tests that reported a positive result for illegal blood alcohol concentration, capturing the population of drivers who were tested [17]  Data is sourced from the Department of Infrastructure, Transport, Regional Development, Communications and the Arts, collated from data collected by multiple organisations. | Proportion of positive Random Breath Tests for alcohol | Estimates were manually calculated based on the number of positive compared to total number of random breath tests. | - Data completeness varies by year and jurisdictions, as detailed in the Road Safety Enforcement Data Dictionary - Data is subject to revision - Organisations collecting data have varied definitions and data collection practices which are not fully consistent across states and territories - In Victoria, the number of random breath tests conducted before 2019 are not reliable - In Tasmania, positive counts of random breath tests were not provided from 2020, so they have been excluded from national calculations - In Western Australia, there was reduced breath testing operations from 2020 and 2021 due to the COVID-19 pandemic |

Abbreviations: AIHW, Australian Institute of Health and Welfare; ICD, International Classification of Diseases; ICD-10, International Classification of Diseases, 10^th^ Revision; ICD-10-AM, International Classification of Diseases, 10^th^ Revision, Australian Modification.

**References**

1.	Australian Institute of Health and Welfare. Alcohol available for consumption in Australia: Technical notes. AIHW Australian Government; 2024. Available from: <https://www.aihw.gov.au/reports/alcohol/alcohol-available-for-consumption-in-australia/contents/technical-notes/background-and-summary>

2.	Australian Criminal Intelligence Commission. *National Wastewater Drug Monitoring Program reports* [Internet]. Canberra: ACIC, 2024. <https://www.acic.gov.au/publications/national-wastewater-drug-monitoring-program-reports>.

3.	Zheng Q, Tscharke B, O'Brien J, Gerber C, Mackie R, Gao J, Thai P. Uncertainties in estimating alcohol and tobacco consumption by wastewater-based epidemiology. Current Opinion in Environmental Science & Health. 2019;9:13-8.

4.	Australian Institute of Health and Welfare. National Drug Strategy Household Survey 2022–2023: Technical notes. AIHW Australian Government; 2025. Available from: <https://www.aihw.gov.au/reports/illicit-use-of-drugs/national-drug-strategy-household-survey/contents/technical-notes>

5.	Wilkins R, Vera-Toscano E, Botha F. The Household, Income and Labour Dynamics in Australia Survey: Selected Findings from Waves 1 to 21. Melbourne Institute: Applied Economic & Social Research, the University of Melbourne; 2024. Available from: <https://melbourneinstitute.unimelb.edu.au/hilda/publications/hilda-statistical-reports>

6.	Australian Bureau of Statistics. *National Health Survey 2022: methodology* [Internet]. Canberra: ABS, 2022. <https://www.abs.gov.au/methodologies/national-health-survey-methodology/2022>.

7.	Australian Bureau of Statistics. 4364.0.55.003 - Australian Health Survey: Updated Results, 2011-2012. Canberra: ABS; 2013. Available from: <https://www.abs.gov.au/AUSSTATS/abs@.nsf/Lookup/4364.0.55.003Explanatory%20Notes12011-2012?OpenDocument>

8.	Australian Institute of Health and Welfare. Alcohol, tobacco & other drugs in Australia: Technical notes. AIHW, Australian Government; 2024. Available from: <https://www.aihw.gov.au/reports/alcohol/alcohol-tobacco-other-drugs-australia/contents/technical-notes>

9.	Chrzanowska A MN, Sutherland R, Degenhardt L, Peacock A. Methods for Trends in overdose and other drug-induced deaths in Australia, 2004-2023. Sydney: National Drug and Alcohol Research Centre, UNSW; 2025. Available from: <https://doi.org/10.26190/unsworks/31246>

10.	Gilmore W, Lensvelt E, Jones P, Dorocicz J, Sherk A, Churchill S, et al. *Australian alcohol-attributable harm visualisation tool: About* [Internet]. National Drug Research Institute, Curtin University and Canadian Institute for Substance Use Research, University of Victoria, 2023. <https://www.alcoholharmtool.info/about.php>.

11.	Sherk A, Stockwell, T., Rehm, J., Dorocicz, J., Shield, K.D. *The International Model of Alcohol Harms and Policies (InterMAHP). Version 2.1* [Internet]. Canadian Institute for Substance Use Research, 2019. <https://www.uvic.ca/research/centres/cisur/projects/intermahp/index.php>.

12.	Australian Institute of Health and Welfare. Alcohol-related injury: hospitalisations and deaths, 2019–20: Methods. AIHW, Australian Government; 2023. Available from: <https://www.aihw.gov.au/reports/injury/alcohol-related-injuries-2019-20/contents/methods>

13.	Chrzanowska A, Man, N, Sutherland, R, Degenhardt, L, Peacock, A. Methods for Trends in drug-related hospitalisations in Australia, 2003-2023. Sydney: National Drug and Alcohol Research Centre, UNSW; 2025. Available from: <https://doi.org/10.26190/unsworks/31345>

14.	Australian Institute of Health and Welfare. Alcohol, tobacco & other drugs in Australia: Alcohol and other drug-related ambulance attendances. AIHW, Australian Government; 2025. Available from: <https://www.aihw.gov.au/reports/alcohol/alcohol-tobacco-other-drugs-australia/contents/data-by-region/ambulance-attendances>

15.	Australian Institute of Health and Welfare. Alcohol and other drug treatment services in Australia annual report. AIHW, Australian Government; 2024. Available from: <https://www.aihw.gov.au/reports/alcohol-other-drug-treatment-services/alcohol-other-drug-treatment-services-australia/contents/about>

16.	Australian Bureau of Statistics. *National, state and territory population* [Internet]. Canberra: ABS, 2025. <https://www.abs.gov.au/statistics/people/population/national-state-and-territory-population/dec-2024>.

17.	Bureau of Infrastructure and Transport Research Economics. *Road safety enforcement data* [Internet]. Department of Infrastructure, Transport, Regional Development, Communications and the Arts, Australian Government, 2024. <https://www.bitre.gov.au/publications/2024/road-safety-enforcement-data>.

## Table S2. Observed values, annual percentage change (APC) and confidence intervals (CI) indicating trends for per capita consumption based on alcohol available for consumption data (sourced from the AIHW) and wastewater estimates (sourced from the NWDMP).

| Year | Total (AIHW) | APC | 95% CIs | Wastewater estimates (NWDMP) | APC | 95% CIs |
| --- | --- | --- | --- | --- | --- | --- |
| 2011 | 10.44 | -1.86 | [-5.12, 1.50] |  |  |  |
| 2012 | 10.14 | -1.86 | [-5.12, 1.50] |  |  |  |
| 2013 | 10 | -1.86 | [-5.12, 1.50] |  |  |  |
| 2014 | 9.92 |  |  |  |  |  |
| 2015 | 9.75 | 0.92* | [0.31, 1.55] |  |  |  |
| 2016 | 10.11 | 0.92* | [0.31, 1.55] | 5.84 | -3.46 * | [-4.42, -2.50] |
| 2017 | 10.1 | 0.92* | [0.31, 1.55] | 5.84 | -3.46 * | [-4.42, -2.50] |
| 2018 | 10.24 | 0.92* | [0.31, 1.55] | 5.84 | -3.46 * | [-4.42, -2.50] |
| 2019 | 10.28 | 0.92* | [0.31, 1.55] | 5.47 | -3.46 * | [-4.42, -2.50] |
| 2020 | 10.04 | 0.92* | [0.31, 1.55] | 5.11 | -3.46 * | [-4.42, -2.50] |
| 2021 | 10.85 | 0.92* | [0.31, 1.55] | 5.11 | -3.46 * | [-4.42, -2.50] |
| 2022 | 10.74 | 0.92* | [0.31, 1.55] | 4.75 | -3.46 * | [-4.42, -2.50] |
| 2023 | 10.46 | 0.92* | [0.31, 1.55] | 4.75 | -3.46 * | [-4.42, -2.50] |
| 2024 |  |  |  |  |  |  |

**indicates significance at p ≤ 0.05 according to modelled trend in Joinpoint analysis. Blank row in APC indicates Joinpoint in data.*

Note: AIHW data reports according to financial years (e.g., 2011-12). For the purposes of table readability, these instances are reported here according to the latest part of the year (e.g., 2012). The denominator for AIHW per capita consumption is persons aged 15 and over. Wastewater numbers are based on author-estimates calculated to litres per person per day; Per capita consumption estimates were calculated using the total estimated catchment population (which includes children).

Abbreviations: AIHW, Australian Institute of Health and Welfare; NWDMP, National Wastewater Drug Monitoring Program.

## Table S3. Observed values, annual percentage change (APC) and confidence intervals (CI) indicating trends in risky drinking (%) among the population, sourced from the NDSHS, NHS and HILDA survey (continued on next page)

| Year | High (weekly) risky drinkers, *15+ (NDSHS)* | APC | 95% CIs | High (monthly) risky drinkers,  *15+ (NDSHS)* | APC | 95% CIs | Long-term  risky drinkers,  *18+ (NHS)* | APC | 95% CIs |
| --- | --- | --- | --- | --- | --- | --- | --- | --- | --- |
| 2010 | 30.9 | -1.83* | [-2.75, -0.90] | 29.4 | -1.57* | [-2.12, -1.02] |  |  |  |
| 2011 |  |  |  |  |  |  |  |  |  |
| 2012 |  |  |  |  |  |  | 19.4 | -3.84* | [-5.65, -1.99] |
| 2013 | 28.3 | -1.83* | [-2.75, -0.90] | 27.4 | -1.57* | [-2.12, -1.02] |  |  |  |
| 2014 |  |  |  |  |  |  |  |  |  |
| 2015 |  |  |  |  |  |  | 17.3 | -3.84* | [-5.65, -1.99] |
| 2016 | 26.6 | -1.83* | [-2.75, -0.90] | 26 | -1.57* | [-2.12, -1.02] |  |  |  |
| 2017 |  |  |  |  |  |  |  |  |  |
| 2018 |  |  |  |  |  |  | 16 | -3.84* | [-5.65, -1.99] |
| 2019 | 25.3 | -1.83* | [-2.75, -0.90] | 25.2 | -1.57* | [-2.12, -1.02] |  |  |  |
| 2020 |  |  |  |  |  |  |  |  |  |
| 2021 |  |  |  |  |  |  |  |  |  |
| 2022 |  |  |  |  |  |  | 12.9 | -3.84* | [-5.65, -1.99] |
| 2023 | 24.9 | -1.83* | [-2.75, -0.90] | 24.1 | -1.57* | [-2.12, -1.02] |  |  |  |

| Year | Single-occasion risky drinkers,  *18+ (NHS)* | APC | 95% CIs | Frequent risky drinkers, *15+ (HILDA)* | | | | | |
| --- | --- | --- | --- | --- | --- | --- | --- | --- | --- |
|  |  |  |  | Males | APC | 95% CIs | Females | APC | 95% CIs |
| 2010 |  |  |  |  |  |  |  |  |  |
| 2011 |  |  |  | 29.5 | -1.40* | [-2.69, -0.09] | 16.5 | -0.62 | [-1.80, 0.57] |
| 2012 | 45.2 | -1.59 | [-4.38, 1.27] |  |  |  |  |  |  |
| 2013 |  |  |  | 26.1 | -1.40* | [-2.69, -0.09] | 15.7 | -0.62 | [-1.80, 0.57] |
| 2014 |  |  |  |  |  |  |  |  |  |
| 2015 | 45 | -1.59 | [-4.38, 1.27] | 25.8 | -1.40* | [-2.69, -0.09] | 16 | -0.62 | [-1.80, 0.57] |
| 2016 |  |  |  |  |  |  |  |  |  |
| 2017 |  |  |  | 26 | -1.40* | [-2.69, -0.09] | 15.6 | -0.62 | [-1.80, 0.57] |
| 2018 | 43.2 | -1.59 | [-4.38, 1.27] |  |  |  |  |  |  |
| 2019 |  |  |  | 25.9 | -1.40* | [-2.69, -0.09] | 16.4 | -0.62 | [-1.80, 0.57] |
| 2020 |  |  |  |  |  |  |  |  |  |
| 2021 |  |  |  | 24.3 | -1.40* | [-2.69, -0.09] | 14.8 | -0.62 | [-1.80, 0.57] |
| 2022 | 37.1 | -1.59 | [-4.38, 1.27] |  |  |  |  |  |  |
| 2023 |  |  |  |  |  |  |  |  |  |

**indicates significance at p ≤ 0.05 according to modelled trend in Joinpoint analysis. Blank row in APC indicates Joinpoint in data.
Definitions: High (weekly) risky drinker: More than 10 standard drinks per week on average in the past 12 months. High (monthly) risky drinker: More than 4 standard drinks on a single day at least once a month over the previous 12 months. Long-term risky drinking: Exceeded guideline “no more than 2 standard drinks per day.” Single-occasion risky drinking: Exceeded guideline “no more than 4 standard drinks on a single occasion.” Frequent risky drinker: Of current drinkers, consume at least 5 (if female) or 7 (if male) standard drinks on one occasion at least 2 or 3 times per month.*
Note: NHS data reports according to financial years (e.g., 2011-12). For the purposes of table readability, these instances are reported here according to the latest part of the year (e.g., 2012). Further, the most recent NDSHS survey wave was 2022-23, but for the purposes of table readability this survey wave is reported here as 2023.

Abbreviations: HILDA, Household, Income and Labour Dynamics in Australia; NDSHS, National Drug Strategy Household Survey; NHS, National Health Survey.

## Table S4. Observed values, annual percentage change (APC) and confidence intervals (CI) indicating trends in deaths related to alcohol (age-standardised rates per 100,000 population), sourced from the AIHW and NAIP.

| Year | Alcohol-related deaths, *entire population (AIHW)* | | | Alcohol-attributable deaths,  *15+ population (NAIP)* | | |
| --- | --- | --- | --- | --- | --- | --- |
|  | Total | APC | 95% CIs | Total | APC | 95% CIs |
| 2010 | 11.9 | 7.03* | [4.73, 9.38] |  |  |  |
| 2011 | 11.6 | 7.03* | [4.73, 9.38] | 17.3 | 3.43* | [3.06, 3.80] |
| 2012 | 11.6 | 7.03* | [4.73, 9.38] | 17.58 | 3.43* | [3.06, 3.80] |
| 2013 | 14.1 | 7.03* | [4.73, 9.38] | 19.1 | 3.43* | [3.06, 3.80] |
| 2014 | 15.3 | 7.03* | [4.73, 9.38] | 19.41 | 3.43* | [3.06, 3.80] |
| 2015 | 15.5 | 7.03* | [4.73, 9.38] | 20.38 | 3.43* | [3.06, 3.80] |
| 2016 | 16.5 | 7.03* | [4.73, 9.38] | 20.81 | 3.43* | [3.06, 3.80] |
| 2017 | 18.3 |  |  | 21.94 | 3.43* | [3.06, 3.80] |
| 2018 | 17.6 | -0.96 | [-4.49, 2.71] | 21.91 | 3.43* | [3.06, 3.80] |
| 2019 | 18.1 | -0.96 | [-4.49, 2.71] | 23.52 | 3.43* | [3.06, 3.80] |
| 2020 | 17.4 | -0.96 | [-4.49, 2.71] | 23.19 | 3.43* | [3.06, 3.80] |
| 2021 | 16.7 | -0.96 | [-4.49, 2.71] | 24.47 | 3.43* | [3.06, 3.80] |
| 2022 | 17.4 | -0.96 | [-4.49, 2.71] | 25.05 | 3.43* | [3.06, 3.80] |
| 2023 |  |  |  |  |  |  |

**indicates significance at p ≤ 0.05 according to modelled trend in Joinpoint analysis. Blank row in APC indicates Joinpoint in data.*

*Definitions: Alcohol-related deaths: Including both deaths directly attributable to alcohol and deaths where alcohol was listed as an associated cause. Alcohol-attributable deaths: Deaths where the underlying cause is either entirely or fractionally caused by alcohol according to calculated Australian-specific alcohol-attributable fractions.*

Abbreviations: AIHW, Australian Institute of Health and Welfare; NAIP, National Alcohol Indicators Project.

## Table S5. Observed values, annual percentage change (APC) and confidence intervals (CI) indicating trends in alcohol-related hospitalisations (per 100,000 population), sourced from the AIHW and NAIP.

| Year | Alcohol-related hospitalisations,  *entire population (NIDIP)* | | | Alcohol-attributable hospitalisations,  *15+ population (NAIP)* | | | Alcohol ambulance attendances,  *entire population (AIHW)* | | |
| --- | --- | --- | --- | --- | --- | --- | --- | --- | --- |
|  |  | APC | 95% CIs | Total | APC | 95% CIs |  | APC | 95% CIs |
| 2011 | 261.64 | 1.30* | [0.66, 1.95] | 468.89 | 1.72* | [1.15, 2.29] |  |  |  |
| 2012 | 265.15 | 1.30* | [0.66, 1.95] | 478.97 | 1.72* | [1.15, 2.29] |  |  |  |
| 2013 | 261.56 | 1.30* | [0.66, 1.95] | 462.93 | 1.72* | [1.15, 2.29] |  |  |  |
| 2014 | 261.14 | 1.30* | [0.66, 1.95] | 467.3 | 1.72* | [1.15, 2.29] |  |  |  |
| 2015 | 261.61 | 1.30* | [0.66, 1.95] | 483.82 | 1.72* | [1.15, 2.29] | 27.12 | 8.57* | [0.46, 17.35] |
| 2016 | 268.05 | 1.30* | [0.66, 1.95] | 503.42 | 1.72* | [1.15, 2.29] | 33.60 | 8.57* | [0.46, 17.35] |
| 2017 | 273.28 | 1.30* | [0.66, 1.95] | 516.38 | 1.72* | [1.15, 2.29] | 34.18 | 8.57* | [0.46, 17.35] |
| 2018 | 275.83 | 1.30* | [0.66, 1.95] | 526.38 | 1.72* | [1.15, 2.29] | 36.83 | 8.57* | [0.46, 17.35] |
| 2019 | 286.89 | 1.30* | [0.66, 1.95] | 533.27 | 1.72* | [1.15, 2.29] | 38.40 |  |  |
| 2020 | 278.3 | 1.30* | [0.66, 1.95] | 519.6 | 1.72* | [1.15, 2.29] | 40.57 | -2.06 | [-13.38, 10.74] |
| 2021 | 323.65 | 1.30* | [0.66, 1.95] | 576.27 | 1.72* | [1.15, 2.29] | 37.67 | -2.06 | [-13.38, 10.74] |
| 2022 | 295.77 | 1.30* | [0.66, 1.95] | 535.5 | 1.72* | [1.15, 2.29] | 37.55 | -2.06 | [-13.38, 10.74] |
| 2023 | 287 | 1.30* | [0.66, 1.95] |  |  |  |  |  |  |

**indicates significance at p ≤ 0.05 according to modelled trend in Joinpoint analysis. Blank row in APC indicates Joinpoint in data.*

*Definitions: Alcohol-related hospitalisations: Hospitalisations where the principal diagnosis was directly attributable to alcohol use. Alcohol-attributable hospitalisations: Hospitalisations based on either primary diagnoses or external causes relating to alcohol use according to calculated Australian-specific alcohol-attributable fractions. Alcohol ambulance attendances: Ambulance attendances with alcohol involvement, identified via paramedic clinical assessment, patient self-report, information from third parties, and/or evidence at the scene.*
Note: The AIHW reports according to financial years (e.g., 2011-12). For table readability, these are reported here according to the later part of the year (e.g., 2012). The AIHW ambulance attendance measure is reported according to the standard year. It should be noted that the ambulance attendance values do not include Western Australia or South Australia, so is not wholly national data.

Abbreviations: AIHW, Australian Institute of Health and Welfare; NAIP, National Alcohol Indicators Project; NIDIP, National Illicit Drug Indicators Project.

## Table S6. Observed values, annual percentage change (APC) and confidence intervals (CI) indicating trends in closed treatment episodes for alcohol (per 100,000 population), sourced from the Australian Institute of Health and Welfare.

| Year | Closed treatment episodes for alcohol | APC | 95% CIs |
| --- | --- | --- | --- |
| 2015 | 318.40 | 4.03* | [2.98, 5.10] |
| 2016 | 322.34 | 4.03* | [2.98, 5.10] |
| 2017 | 312.87 | 4.03* | [2.98, 5.10] |
| 2018 | 349.45 | 4.03* | [2.98, 5.10] |
| 2019 | 362.77 | 4.03* | [2.98, 5.10] |
| 2020 | 359.01 | 4.03* | [2.98, 5.10] |
| 2021 | 399.07 | 4.03* | [2.98, 5.10] |
| 2022 | 410.89 | 4.03* | [2.98, 5.10] |
| 2023 | 422.39 | 4.03* | [2.98, 5.10] |

**indicates significance at p ≤ 0.05 according to modelled trend in Joinpoint analysis. Blank row in APC indicates Joinpoint in data.*

Note: Australian Institute of Health and Welfare treatment data reports according to financial years (e.g., 2014-15). For the purposes of table readability, these instances are reported here according to the latest part of the year (e.g., 2015). Definition of closed treatment episode: Treatment for one’s own use where alcohol was listed as the principal drug of concern, determined due to being the main treatment type, reason for cessation, or source of referral.

## Table S7. Observed values, annual percentage change (APC) and confidence intervals (CI) indicating trends in the proportion (%) of positive random breath tests for alcohol, sourced from the Bureau of Infrastructure and Transport Research Economics

| Year | Positive random breath test for alcohol (%) | APC | 95% CIs |
| --- | --- | --- | --- |
| 2010 | 0.81 | -8.12* | [-13.79, -2.07] |
| 2011 | 0.73 | -8.12* | [-13.79, -2.07] |
| 2012 | 0.64 | -8.12* | [-13.79, -2.07] |
| 2013 | 0.62 | -8.12* | [-13.79, -2.07] |
| 2014 | 0.56 | -8.12* | [-13.79, -2.07] |
| 2015 | 0.54 | -8.12* | [-13.79, -2.07] |
| 2016 | 0.49 | -8.12* | [-13.79, -2.07] |
| 2017 | 0.45 |  |  |
| 2018 | 0.43 | 8.25 | [-2.70, 20.43] |
| 2019 | 0.43 | 8.25 | [-2.70, 20.43] |
| 2020 | 0.76 | 8.25 | [-2.70, 20.43] |
| 2021 | 0.65 | 8.25 | [-2.70, 20.43] |
| 2022 | 0.56 | 8.25 | [-2.70, 20.43] |

**indicates significance at p ≤ 0.05 according to modelled trend in Joinpoint analysis. Blank row in APC indicates Joinpoint in data.*

Note: During 2020-2022 inclusive, Tasmania did not provide data for random breath tests.
